# Bi-directional and shared epigenomic signatures following proton and ^56^Fe irradiation

**DOI:** 10.1038/s41598-017-09191-4

**Published:** 2017-08-31

**Authors:** Soren Impey, Timothy Jopson, Carl Pelz, Amanuel Tafessu, Fatema Fareh, Damian Zuloaga, Tessa Marzulla, Lara-Kirstie Riparip, Blair Stewart, Susanna Rosi, Mitchell S. Turker, Jacob Raber

**Affiliations:** 1 Oregon Stem Cell Center and Department of Pediatrics, Oregon Health and Science University, Portland, OR 97239 USA; 2 Department of Cell and Developmental Biology, Oregon Health and Science University, Portland, OR 97239 USA; 3 Brain and Spinal Injury Center, Departments of Neurological Surgery and Physical Therapy and Rehabilitation Science, University of California, San Francisco, San Francisco, CA 94110 USA; 4 Department of Behavioral Neuroscience, Oregon Health and Science University, Portland, OR 97239 USA; 5 Oregon Institute of Occupational Health Sciences and Department of Molecular and Medical Genetics, Oregon Health and Science University, Portland, OR 97239 USA; 6 Departments of Neurology and Radiation Medicine, Division of Neuroscience ONPRC, Oregon Health and Science University, Portland, OR 97239 USA

## Abstract

The brain’s response to radiation exposure is an important concern for patients undergoing cancer therapy and astronauts on long missions in deep space. We assessed whether this response is specific and prolonged and is linked to epigenetic mechanisms. We focused on the response of the hippocampus at early (2-weeks) and late (20-week) time points following whole body proton irradiation. We examined two forms of DNA methylation, cytosine methylation (5mC) and hydroxymethylation (5hmC). Impairments in object recognition, spatial memory retention, and network stability following proton irradiation were observed at the two-week time point and correlated with altered gene expression and 5hmC profiles that mapped to specific gene ontology pathways. Significant overlap was observed between DNA methylation changes at the 2 and 20-week time points demonstrating specificity and retention of changes in response to radiation. Moreover, a novel class of DNA methylation change was observed following an environmental challenge (i.e. space irradiation), characterized by both increased and decreased 5hmC levels along the entire gene body. These changes were mapped to genes encoding neuronal functions including postsynaptic gene ontology categories. Thus, the brain’s response to proton irradiation is both specific and prolonged and involves novel remodeling of non-random regions of the epigenome.

## Introduction

A unique feature of the space radiation environment is the presence of galactic cosmic rays (GCR) and solar particle events (SPE)^1^. The former involves protons and fully ionized atomic nuclei while the latter includes predominantly low to medium energy protons^2^. Proton radiation exposures may pose a significant health hazard to space flight crews during and following the mission^3^. Proton irradiation is also clinically relevant, as it is increasingly used in cancer therapy^4–6^. The hazards associated with the space environment will likely impact many organ systems. In the central nervous system (CNS), radiation exposure significantly affects the hippocampus^7–24^, a structure critical for memory function. For example, object recognition memory^25^, which uses a 24-hour interval between learning and memory assessment to test hippocampal function^26^, is impaired twelve weeks following irradiation of 2-month-old mice with protons (150 MeV, 0.1 Gy)^27^. Young-adult mice exposed to protons (3 and 4 Gy, 250 MeV) failed to show habituation to an open field when tested two weeks after exposure^28^. Similarly, spatial learning and memory in the water maze were not affected by proton irradiation of young-adult (250–275 g) rats (1.5, 3, or 4 Gy, 250 MeV)^29^. Whether habituation to an open field, object recognition, and spatial learning and memory in the water maze are sensitive to effects of proton irradiation in adult mice irradiated at 6 months of age, a biological age relevant to the typical ages of astronauts during space missions, is not known.

The mechanisms mediating the effects of proton irradiation on hippocampus-dependent cognitive function might be associated with changes in hippocampal networks involved in synaptic plasticity and memory. Age-related changes in immediate early gene Activity-Regulated Cytoskeleton-Associated Protein (*Arc*) in the hippocampus have been reported^30^. The study of *Arc* expression provides important insight into the post-transcriptional infrastructure of gene expression involved in synaptic plasticity and memory^31^ (for review, ref. 32). When neurons are engaged in information processing, Arc is rapidly transcribed and can be visualized and quantified after ~5 min. Subsequently, the mRNA is translocated to the cytoplasm where it remains detectable for ~20–30 min after the initial transcription. Thus, two different cellular compartments (nuclear and cytoplasmic) can be clearly distinguished, providing means to identify which neurons were active during distinct behavioral experiences^31^. Analysis of the temporal kinetics of *Arc* mRNA allows study of neuronal activity and network stability by detecting the number of active cells forming environment-specific maps and capturing the environmental specificity of place fields^31, 33–35^.

Hippocampal DNA methylation is affected 22 weeks following proton irradiation^36^ and might be involved in short- and long-term effects of proton irradiation on hippocampus-dependent cognitive performance. Hippocampal changes in cytosine methylation, a major epigenetic modification involving the addition of a methyl group to cytosine (5mC), play a key role in regulating expression of genes required for spatial learning and memory^37, 38^. A second form of DNA methylation, hydroxymethylcytosine (5hmC), is derived from 5mC by the action of three TET enzymes (TET 1–3)^39^. In brain, levels of TET2 are higher than those of TET1 or TET3 and therefore TET2 is believed most important for brain function^40, 41^. The levels and localizations of 5mC and 5hmC levels are high and exceptionally dynamic in the brain^42–44^. Their high and dynamic levels in neurons during development and aging^45, 46^ suggest strongly that they play critical roles.

In the current study, we assessed the effects of whole body proton irradiation at early (2-week) and late (20-week) time points on hippocampus-dependent cognitive performance and whether these effects are associated with changes in hippocampal DNA methylation (both 5mC and 5hmC).

## Methods and Materials

### Animals and Study Design

Six-month-old C57BL/6 J male mice (*n* = 84 mice in total) were obtained from Jackson Laboratories, Bar Harbor Maine. The biological age of the mice was selected to be relevant to the biological age of astronauts during space missions. The mice were shipped from Jackson Laboratories to Brookhaven National Laboratory (BNL), Upton, New York, and allowed to accommodate to the housing facility there for one week. Subsequently, the mice were irradiated with 1 Gy of 150 MeV protons or sham-irradiated (*n* = 42 mice/radiation dose). For irradiation, mice were individually loaded into 8 × 3 × 3 cm plastic square enclosures with air holes and placed in a foam fixture in the beam line of the NASA Space Radiation Laboratory (NSRL). They were exposed to a rectangular beam of approximately 20 × 20 cm. The focused beam of high-energy was generated by the Booster accelerator at BNL and transferred to the experimental beam line at the NSRL facility. Dose calibration was performed so that the desired dose could be delivered. Sham-irradiated mice were placed into the plastic enclosures for the same amount of time as the irradiated mice. Mice were randomly assigned to the experimental groups. The week after the irradiation or sham-irradiation, the mice were shipped to Oregon Health & Science University (OHSU) and were assigned to one of two time points (2 or 20 weeks; *n* *=* 42 mice/time point). Cognitive testing started two or twenty weeks following irradiation. Following cognitive testing, the mice were killed by cervical dislocation followed by decapitation. The hippocampus of one hemibrain was dissected for DNA methylation and RNAseq analyses. The other hemibrain was processed for *Arc* mRNA and TET2 immunohistochemical analyses. All protocols were reviewed and approved by the Institutional Animal Care and Use Committees (IACUC) of OHSU and BNL and were in compliance with all Federal regulations.

### Novel Object Recognition

The novel object recognition test was performed as described^47^. The mice were habituated to an open field (16 × 16 inches, Kinder Scientific, Poway, CA) for 3 times for 10 min each over three subsequent days. On day 4, the mice were placed in the open field containing two identical objects and they were allowed to freely explore for 15 minutes. On day 5, the mice were placed again in the open field, but one familiar object was replaced with a novel object. The mice were allowed to explore for 15 minutes. Movement and time spent exploring each object was recorded using Ethovision XT video tracking system (Noldus Information Technology, Sterling, VA) and hand scored by a researcher blinded to the treatment of the mice. The percent time exploring the novel object, out of the total time exploring the novel and familiar objects on day 5, was used to assess novel object recognition. For each group, the preference for the novel versus the familiar object was assessed. The open field arena and objects were cleaned with 5% acetic acid between mice and trials.

### Water Maze

Spatial learning and memory was assessed in the water maze as described^47^. A circular pool (diameter 140 cm) was filled with water made opaque with nontoxic chalk (24 °C) and mice were trained to locate a submerged platform. To determine if irradiation affected the ability to swim or learn the water maze task, mice were first trained to locate a clearly marked platform (visible platform, Days 1 and 2). The mice were subsequently trained to locate the platform when it was hidden beneath the surface of opaque water (Days 3–5). Training during the hidden platform sessions (acquisition) required the mice to learn the location of the hidden platform based on extra-maze cues. For both visible and hidden sessions, there were two daily sessions, morning and afternoon, which were 2-h apart. Each session consisted of two trials (with 5-min inter-trial intervals). A trial ended when the mice located the platform. Mice that failed to locate the platform within 60 s were led to the platform by placing a finger in front of their swim path. The mice were taken out of the pool after they were physically on the platform for a minimum of 3 s. During visible platform training, the platform was moved to a different quadrant of the pool for each session. For the hidden platform training, the platform location was kept constant. The mice were placed into the water facing the edge of the pool in one of nine randomized locations. The start location was changed for each trial. The swimming patterns of the mice were recorded with Noldus Ethovision video tracking software (Ethovision XT, Noldus Information Technology, Wageningen, Netherlands) set at six samples/s. The time to locate the platform (latency) and cumulative distance to the target were used as measures of performance for the visible and hidden sessions. Because swim speeds can influence the time it takes to reach the platform, they were also analyzed.

To measure spatial memory retention, probe trials (platform removed) were conducted 24 h after the last hidden trial of each mouse on the second day of hidden platform training and 72 h following the last hidden trial of each mouse on the third day of hidden platform training and the time spent in the target quadrant as compared to any other quadrant during the probe trials was analyzed.

### Hippocampal network stability

Three days after the last water maze test day, exploration of identical or different environments was used to study the stability of hippocampal networks^31^. The method called catFISH (cellular compartment analysis of temporal activity using fluorescence *in situ* hybridization) relies on the precise temporal kinetics of the IEG *Arc*, which has been used to provide important insight into the post-transcriptional infrastructure of gene expression involved in synaptic plasticity and memory^31, 33^, (for review, ref. 32). When neurons are engaged in information processing, *Arc* is rapidly transcribed and can be visualized and quantified after ~5 min. Subsequently, the mRNA is translocated to the cytoplasm where it remains detectable for ~20–30 min after the initial transcription. Ultimately, the mRNA is translocated to tagged synapses for protein synthesis. Thus, two different cellular compartments (nuclear and cytoplasmic) can be clearly distinguished, providing means to identify which neurons were active during distinct behavioral experiences^31^.

Eight mice from each experimental radiation condition were placed individually into a novel environment (A) and allowed to explore for five minutes, as described^33^. Environment A is a square open field (61 × 61 cm box with 20-cm high walls). After exploration, mice were returned to their cage for 25 min, returned to the same environment for an additional 5 min (AA Paradigm). Another 8 mice of each experimental radiation condition were allowed to explore environment A for 5 minutes, and 25 minutes later they were placed in a different environment (B), a circular arena 45 cm in diameter, and allowed to explore for 5 minutes (AB Paradigm). Following the last environmental exposure, the mice were killed by cervical dislocation and the brains quickly removed, as described above.

Using catFISH, Arc mRNA appear as discrete nuclear foci (recent transcription ~5–10 min), and/or as diffuse mRNA in the cytoplasm (earlier transcription). Nuclear and cytoplasmic *Arc* can be distinguished using intronic and full-length probe respectively labeled with digoxigenin or fluorescein. *Arc* staining was classified as: a) None (no *Arc* staining); b) cytoplasmic *Arc* staining only; c) nuclear *Arc* staining only; or d) both *Arc*-foci/*Arc*-cyto (containing both foci and cytoplasmic staining). We can determine if the neurons responding (i.e. *Arc*+) to an initial experience (exploration of environment A) are the same neurons that respond to a second and identical experience as expected based on sham-irradiated animals (AA Paradigm), and if the hippocampal networks activated during exploration of the first environment are statistically independent from those activated by the exploration of a different environment (AB Paradigm). For the CA1 and CA3 regions of the hippocampus, the percentage of neurons showing both foci and cytoplasmic staining was analyzed. As the pattern of percentages of *Arc*-positive neurons in both environments might reflect differences in the total number of *Arc*-positive neurons (total number of neurons with *Arc* nuclear foci, cytosolic *Arc*, or both) this number was also determined. For the dentate gyrus, the percent of neurons expressing *Arc* only in the cytoplasm or expressing both *Arc*-foci/*Arc*-cyto (containing both foci and cytoplasmic staining) was analyzed. This measure was analyzed as it reflects the total response to the environment(s).

### Tet2 Immunohistochemistry

Following cervical dislocation and decapitation, hemibrains were rapidly removed and frozen using isopentane and dry ice, as described^48^ and stored at −80 °C. The hemibrains were shipped to the University of California, San Francisco, and cut at 20 µm using a cryostat. Slides were sent to OHSU for analysis of Tet2 immunoreacttivity using a specific primary antibody from Santa Cruz Biotechnology (Tet2 S-13, catalog number sc-136926). Briefly, sections (*n* = 3 sections per hemibrain and approximately 200 µm apart) were rinsed in phosphate buffered saline (PBS), and incubated in 4% normal goat serum (NGS) in PBS with 0.4% triton X-100 (PBS-TX). Next, sections were incubated overnight with primary antibodies against Tet2 (1:250) in 4%NGS in PBS-TX. The next day, tissue sections were washed in PBS, incubated for 2.5 hours in donkey anti-rabbit Alexa 488 (1:200) in 4% NGS in PBS-TX and again rinsed in PBS. Analysis of Tet2 immunoreactivity was performed using an Olympus IX81 confocal microscope equipped with Slidebook software. Images of hippocampal regions (CA1, CA3, and dentate gyrus) and the cortex were captured within 3 sections (Bregma −1.58 to −2.46) using a 20x objective (UPlan FL, Olympus). Tet2 immunoreactivity was quantified within fixed area frames; CA1 (box, 125 × 50 µm), CA3 (2 boxes, 95 × 85 µm each), dentate gyrus (2 boxes, 95 × 85 µm each), and cortex (posterior parietal association area; box, 240 × 200 µm). Background threshold levels were set and applied to all images for comparison. Pixel intensities above this threshold were used for quantification measures (area occupied by pixels and intensities of pixels). The total intensity was also quantified as a measure of overall pixel intensity within a specific brain region.

### DNA methylation

DNA was isolated from the hippocampus. For the 2-week time point, antibodies against 5mC and 5hmC were used to immunoprecipitate sonicated DNA preparations for methyl-DNA immunoprecipitation (meDIP–anti-5mC mouse mAb, EMD-Millipore NA8; catalog number 162 33 D3) and hydroxymethyl-DNA immunoprecipitation (hmeDIP–anti-5hmC rabbit polyclonal – Active Motif; catalog number 39769), respectively, from eight pools of tissues (4 × 2 pools of hippocampal tissues or 2 pools/radiation condition/time point). For the 20-week time point, the isolation of the DNA and DNA methylation was analyzed using 2 × 2 pools of hippocampal tissues, as reported^36^. These antibodies were used to precipitate genomic regions that are enriched for either 5mC or 5hmC. Following immunoprecipitation, high throughput genomic sequencing was used to identify the enriched genomic regions. For DIP-Seq library preparation, RNAse-treated DNA was isolated using the Qiagen Allprep DNA/RNA protocol. The DNA was sonicated using a Cole Parmer CPX-132 sonicator (75% amplitude, 3 × 10′) and polished using the DNA terminator end repair kit (Lucigen). DNA fragments were A-tailed using Klenow exo- (Epicenter) and ligated to un-methylated HT TrueSeq indexed adapters and purified. The resulting purified DNA was denatured at 95 C, resuspended in 100 ul of DIP IP buffer, and immunoprecipitated with 1 μg of the highly specific 5-methylcytosine antibody (Eurogentec) or 2 ul of 5-hydroxymethylcytosine (Active Motif) antibody and Dynal anti-mouse IgG beads. Beads were rinsed 7 times with IP buffer, eluted with 1% SDS at room temperature and the eluted DNA purified and subjected to limited amplification (~18 cycles). Libraries were sequenced on the HiSeq. 2000 platform at the OHSU Massively Parallel Sequencing Shared Resource or the Oregon State University Center for Genome ReseArch. DIP-Seq regions methylated above “background” were identified using a sliding window method and enriched regions selected via a Montecarlo-permutation test^49^.

### RNAseq

To facilitate direct comparison of DIP-Seq data with gene expression data for the 2-week time point, RNA-Seq was used to profile transcription from the same animals used for the DIP-Seq experiments. For the 2-week time point, RNA was isolated using the NEBnext poly A selection kit (New England Biolabs). Illumina high-throughput sequencing technology was used to profile RNA levels in an unbiased manner. Differential methylation may occur at novel regions and the unbiased nature of RNA-Seq analyses enables analysis of associated un-annotated transcription. For Illumina RNA-Seq library preparation, we used the NEBnext Ultra kit according to the manufacturers specifications (New England Biolabs). Libraries were sequenced on the HiSeq. 2000 platform at the OHSU Massively Parallel Sequencing Shared Resource. Illumina data were mapped to the UC Santa Cruz assembly using Bowtie^50^. Read statistics and quality control data for sequencing data can be found in Supplemental Table 1 and Supplemental Figs 1–3. For RNA-Seq analyses, tags that overlap with known RefSeq gene models (UCSC RefSeq annotation) were counted using R scripts^51^. Significance was assessed using the DESeq2 package^52^. The Storey Q-test was used to adjust for multiple comparisons^53^. Differential exon usage analyses utilized Tophat gapped alignment^54^ and the DEXSeq pipeline as described in the supplemental materials^55^.

### Bioinformatics and statistical analyses

All behavioral, cognitive, and *Arc* data are shown as mean ± SEM. The statistical analyses of the data were performed using SPSS™ (Chicago, IL) and GraphPad Prism™ (San Diego, CA) software packages. To assess effects of radiation on spatial memory retention in the individual probe trials, ANOVAs were used, followed up by Dunnett’s posthoc tests when appropriate. To analyze locomotor activity over three days, ability to locate a visible platform over two days, and ability to locate a hidden platform over three days, repeated measures ANOVA was used. To compare exploration of the objects and the percentage of *Arc*-positive cells and total number of *Arc* cells following exposure to the two different environmental conditions, t-tests were used. All figures were generated using GraphPad Prism software. We considered *p* < 0.05 as statistically significant.

Single read sequence data was mapped to the mouse reference genome (UCSC mm9) using the Bowtie algorithm using standard flags and allowing 2 mismatches^50^. Sequences that map to a single location were selected and domains enriched for 5mC or 5hmC were selected using a parameter-optimized Monte-Carlo-based segmentation algorithm^49^. A 1000 bp sliding-window was used based on iterative analyses that maximized the number of enriched regions. A comparison of different high-throughput sequencing based methods to study DNA methylation concluded that MeDIP-Seq covers ~67% of genomic CpGs^56^.

For statistical comparisons of biological samples, regions of methylation enrichment were merged and differences in methylation interrogated with FDR-adjusted negative binomial statistics^57^. Statistical and visualization studies involved the R programming language and Bioconductor packages^51^. Gene ontology analyses utilized the Bioconductor Goseq package, which adjusts for RNA-Seq length bias artifacts^58^. KEGG analyses were conducted using the DAVID-EASE site^59^. For gene ontology analyses the top 2000 DMRs (differentially methylation regions) or DHRs (differentially hydroxymethylated regions) (FDR-adjusted *p* < 0.01) within a 50 kb window centered on the transcriptional start site were non-redundantly annotated. Unless otherwise stated, overlap between DMRs and RNA-Seq data was analyzed using a similar windowing approach.

DIP sequence-tag heatmaps were generated in R by plotting median-normalized DIP-Seq tag density in gene bodies and indicated flanking regions with color-maps scaled to the 80% quantile. Statistical analyses of pathway data were conducted via FDR-adjusted Fisher exact test. Statistical analyses of DHR density in genomic regions were conducted using a Monte-Carlo-based permutation statistic (“coin” R package). We considered (FDR-adjusted) *p* < 0.01 as statistically significant.

## Results

### Effects of proton irradiation on cognitive performance at the two-week time point

**Figure 1:**
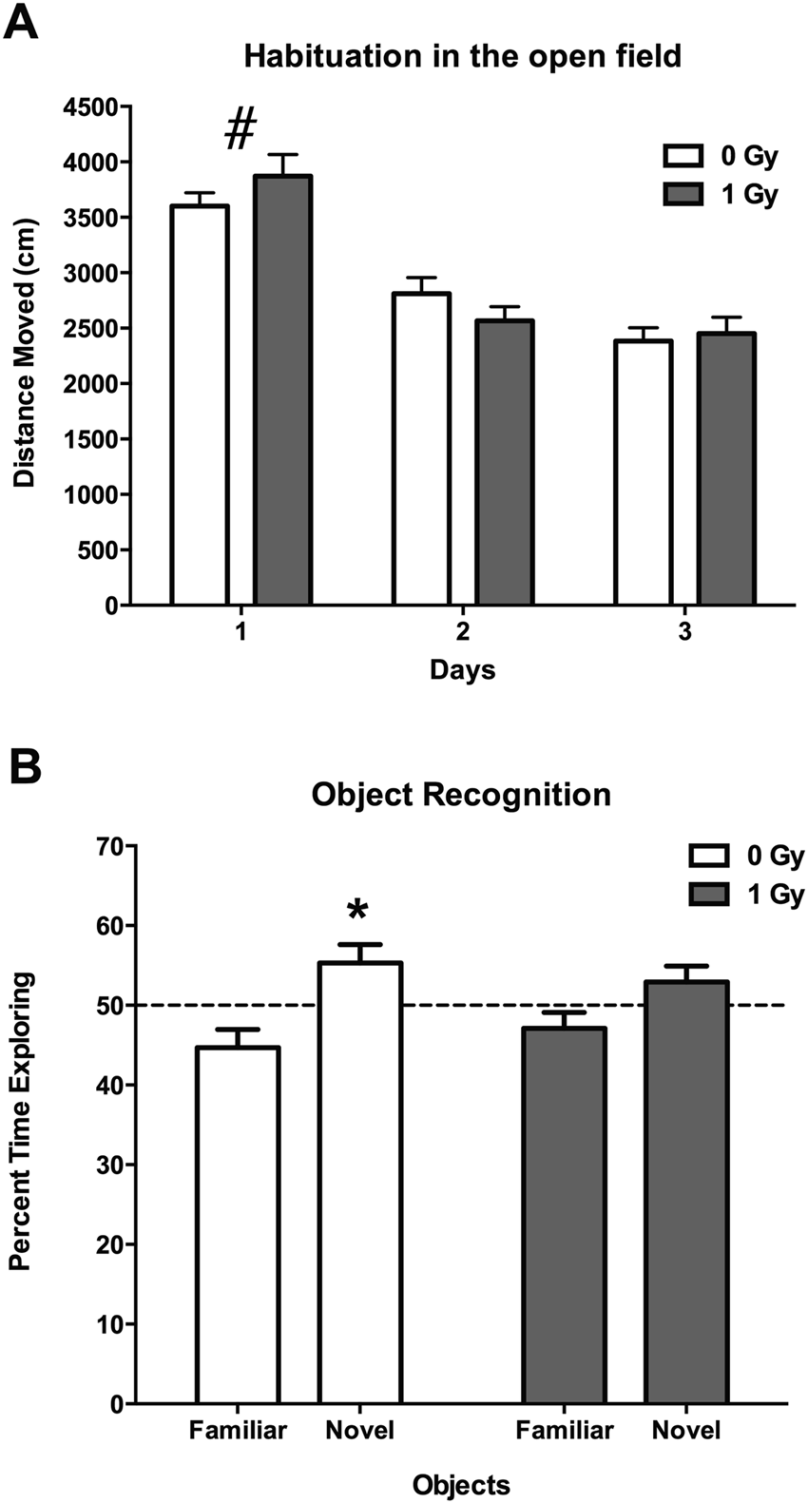
(**A**) Habituation of sham-irradiated and proton-irradiated mice at the 2-week time point. There was an effect of day (*F* = 140.6, *p* < 0.0001), indicating that both groups habituated to the open field. ^#^ *p* < 0.0001 versus days 2 and 3. (**B**) Object recognition at the 2-week time point. Sham-irradiated showed object recognition and spent significantly more time exploring the novel than the familiar object. In contrast, irradiated mice did not. *N* = 16 mice/dose. *p < 0.05.

When activity levels in the open field over three days were analyzed, sham-irradiated and irradiated mice habituated to the open field and showed higher activity levels in the open field on the first day than on subsequent days (effect of day: *F* = 140.6, *p* < 0.0001; Fig. 1A). In addition, there was a day × radiation interaction (*F* = 4.833, *p* = 0.01), but when activity levels were analyzed for each of the three days separately there were no significant effects of irradiation on activity levels. However, detrimental effects of proton irradiation were seen when object recognition was assessed. While sham-irradiated mice showed object recognition and spent significantly more time exploring the novel than the familiar object (*t* = 2.319, *p* < 0.05), irradiated mice did not (*t* = 1.439, *p* = 0.1708) (Fig. 1B).

**Figure 2:**
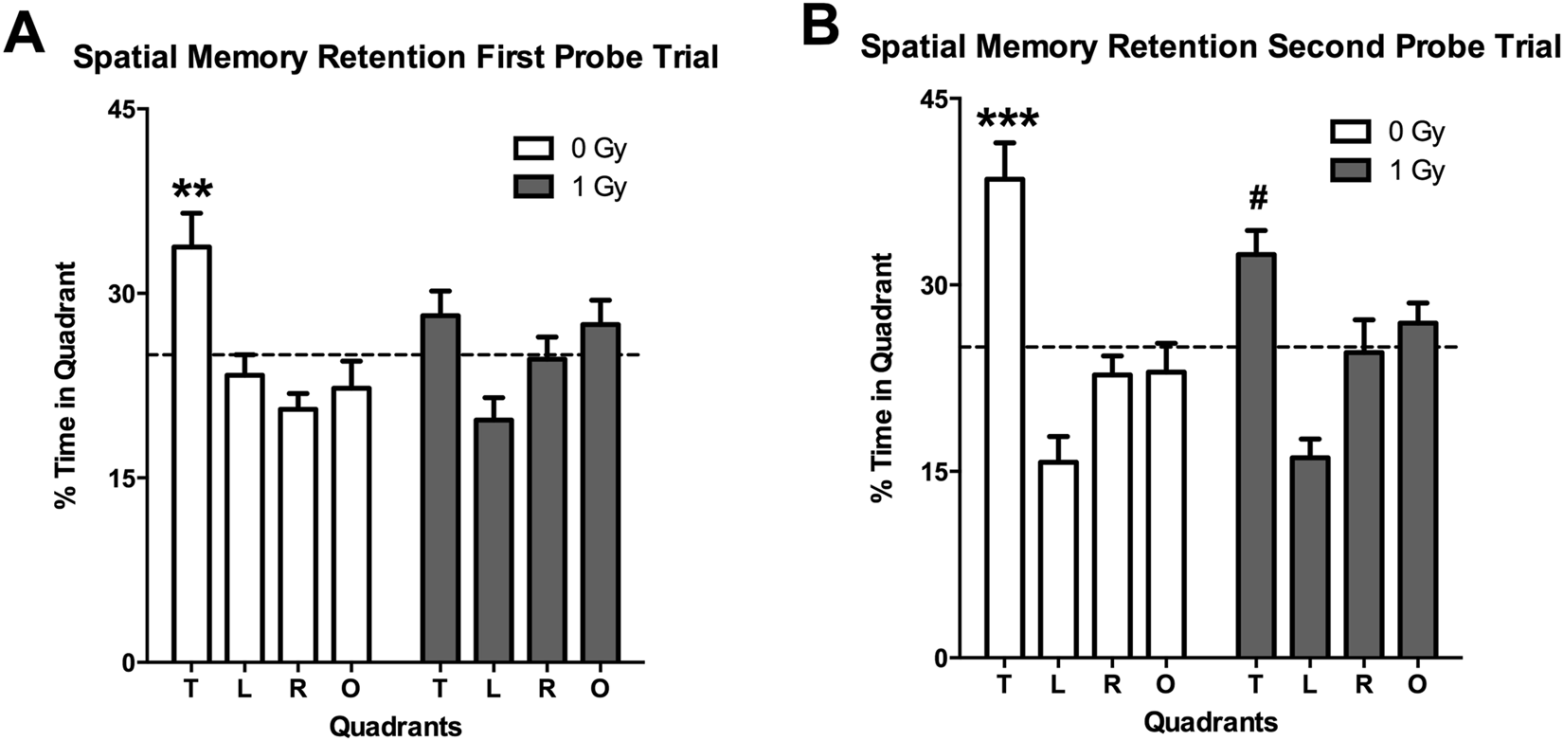
(**A**) Performance in first water maze probe trial at the 2-week time point. Sham-irradiated mice spent more time in the target quadrant than any other quadrant (effect of quadrant: *F* = 6.356, *p* = 0.001) but proton irradiated mice did not. (**B**) Performance in second water maze probe trial at the 2-week time point. Sham-irradiated mice spent more time in the target quadrant than any other quadrant (effect of quadrant: *F* = 13.47, *p* < 0.0001) but proton irradiated mice did not. *N* *=* 16 mice/dose. **Target versus left and opposite quadrants: p < 0.01; Target versus right quadrant p < 0.001; ***Target versus any other quadrant: *p* < 0.001. ^#^Target versus right quadrant: *p* < 0.05; target versus left quadrant: *p* < 0.001.

When mice were trained to locate a visible and subsequent hidden platform in the water maze, both groups improved their performance with training (effects of session: *F* = 14.14, *p* < 0.0001) but there was no effect of radiation (not shown). However, effects of proton irradiation were seen when spatial memory retention was assessed in the first probe trial (no platform) 24 hours after the second day of hidden platform training. Sham-irradiated mice showed spatial memory retention and spent more time in the target quadrant that had contained the platform during the hidden platform sessions, but proton-irradiated mice did not (Fig. 2A). However, this impairment was not seen following additional training. In the second probe trial, 72 hours following the third day of hidden platform training sham-irradiated and irradiated mice both showed spatial memory retention and spent more time in the target quadrant than any other quadrant (Fig. 2B).

### Effects of proton irradiation on network stability in the hippocampus at the two-week time point

**Figure 3:**
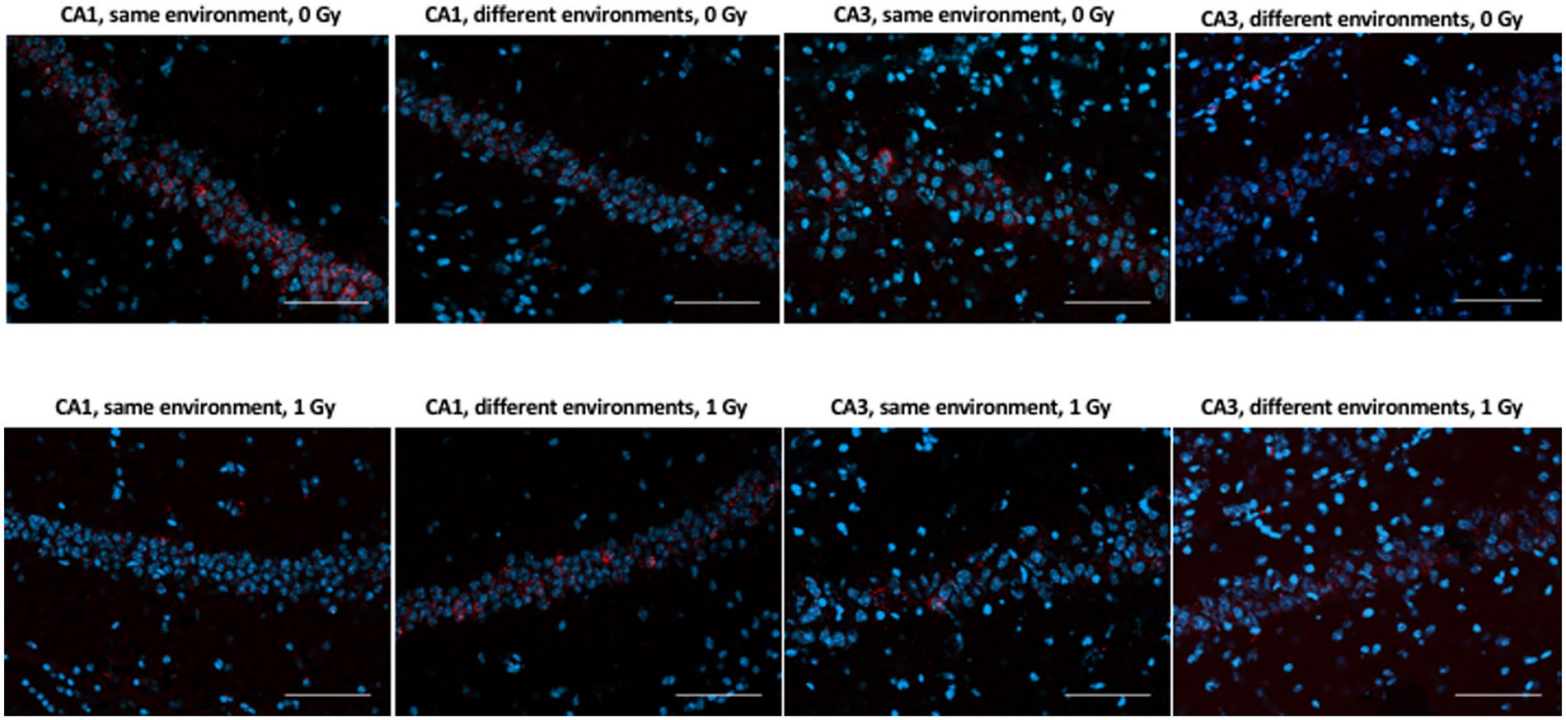
Representative images for *Arc* catFISH data in areas CA1 and CA3 regions of the hippocampus. Representative fluorescence images showing Arc mRNA expression following exploration of the same or different environments (images taken at 20 × 1 z stack). Scale bar: 100 μm. The *Arc* mRNA is illustrated in red and cell nuclei are indicated in blue (DAPI).

**Figure 4:**
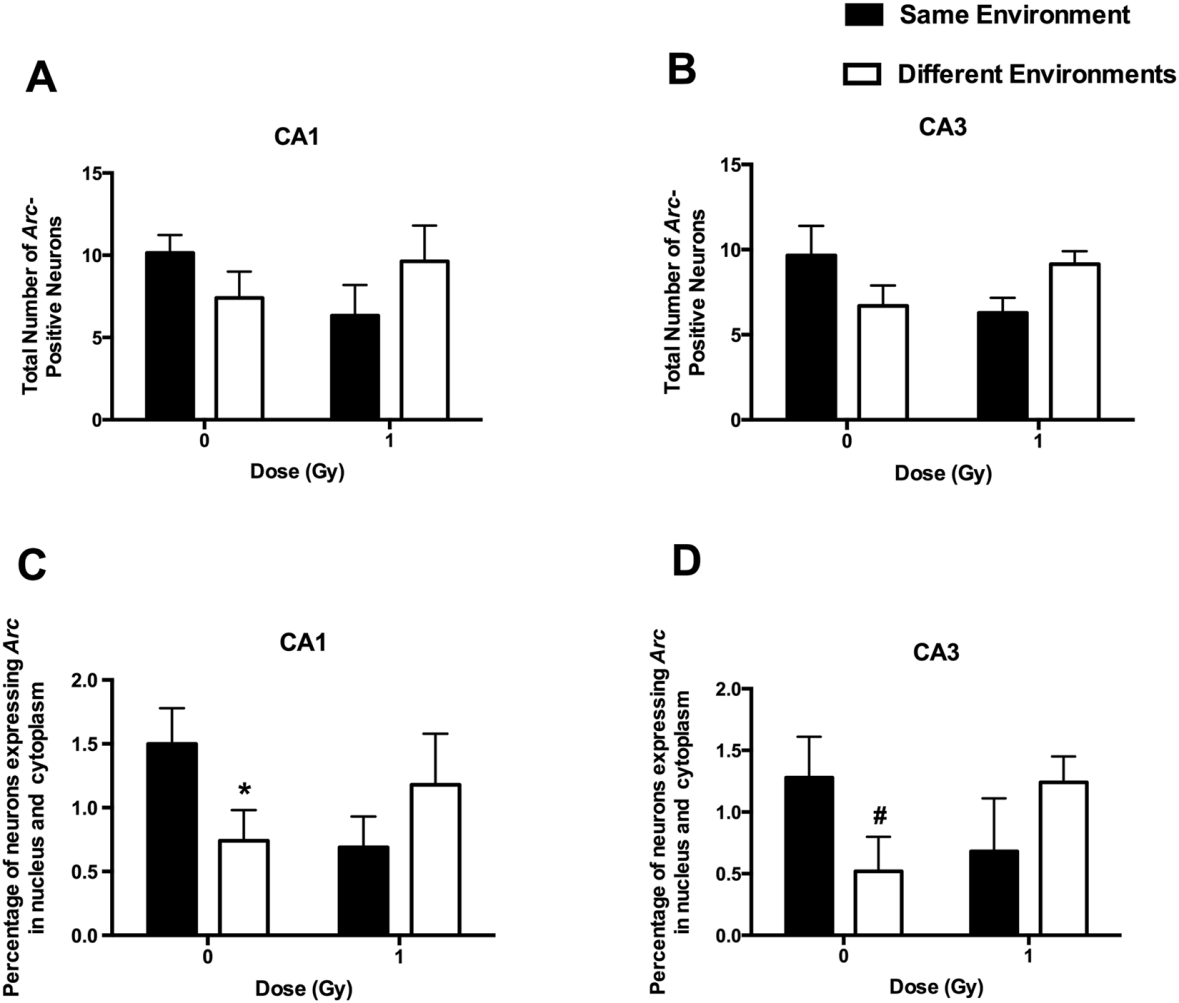
A comparable total number of *Arc*-positive neurons in the CA1 (**A**) and CA3 (**B**) regions of the hippocampus at the 2-week time point. The percentage of *Arc*-positive neurons in the CA1 region of the hippocampus at the 2-week time point. (**C**) The percentage of *Arc*-positive neurons in the CA1 region of the hippocampus of sham-irradiated mice was higher following exposure to twice the same environment than two different environments. This was not seen in irradiated mice. (**D**) There was a trend towards a higher percentage of *Arc*-positive neurons expressing *Arc* mRNA in the nucleus and cytoplasm in the CA3 region of the hippocampus following exposure twice to the same environment, as opposed to exposure to two different environments in sham-irradiated mice but that did not reach significance. *p < 0.05 versus same environment; ^#^ *p* *=* 0.0578. *N* = 5–7 mice/dose/environment.

To determine whether the cognitive impairments were associated with reduced ability of hippocampal neurons to recognize similar environments and to discriminate distinct environments, we assessed the effects of proton irradiation on network stability. This ability was analyzed by assessing the percentage of *Arc*-positive neurons expressing both *Arc*-positive foci in the nucleus and *Arc* staining in the cytoplasm. This combination represents the population of neurons activated by both experiences (See Fig. 3 for representative images of all eight experimental groups). Exploratory behavior activated a comparable total number of neurons in sham-irradiated and irradiated mice in the CA1 (Fig. 4A) and CA3 (Fig. 4B) regions of the hippocampus. However, there was an effect of irradiation on the percent of neurons activated by both experiences. The percentage of *Arc*-positive neurons expressing *Arc* mRNA in the nucleus and cytoplasm in the CA1 region of the hippocampus of sham-irradiated mice was significantly higher following exposure twice to the same environment, as opposed to exposure to two different environments (*t* *=* 2.014, *p* = 0.0374), but this was not seen in irradiated mice (*t* *=* 1.046, *p* = 0.1502) (Fig. 4C). In addition, there was a trend towards a higher percentage of *Arc*-positive neurons expressing *Arc* mRNA in the nucleus and cytoplasm in the CA3 region of the hippocampus following exposure twice to the same environment, as opposed to exposure to two different environments in sham-irradiated mice but that difference was just short of significance (*t* = 1.796, *p* *=* 0.0578, Fig. 4D). No trend was seen in irradiated mice, which demonstrates that the discrimination observed between the same and different environments in the control mice was lost after proton irradiation.

### Effects of proton irradiation on DNA methylation in the hippocampus at the two-week time point

**Figure 5:**
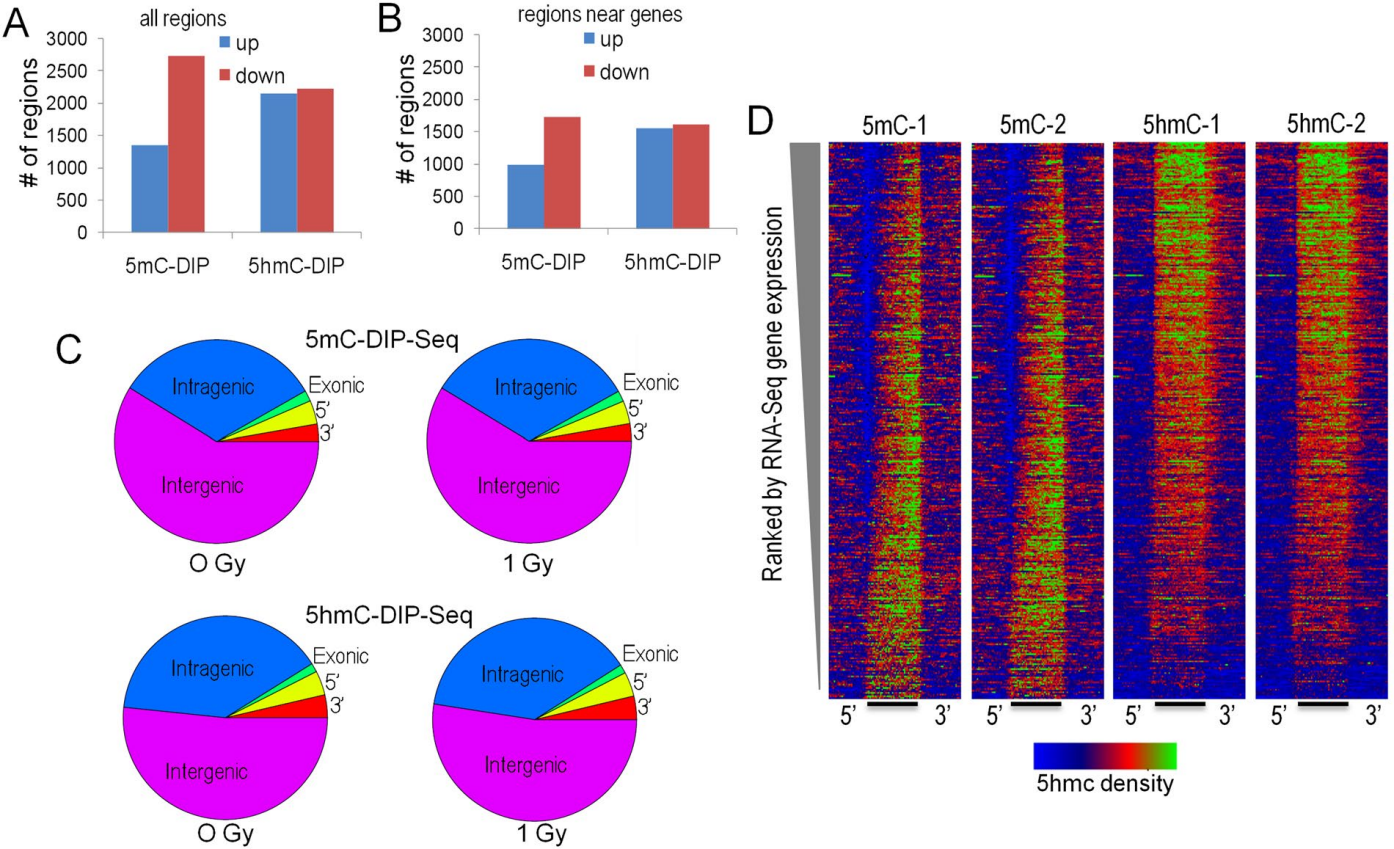
(**A**) Number of regions with significantly increased (blue) and decreased (red) 5hmC and 5 mC. (**B**) Number of significantly-regulated regions within 25 kb of a RefSeq gene start site. (**C**) Venn Diagrams depicting the annotation of differentially methylated and hydroxymethylated regions relative to gene boundaries. (**D**) DIP-Seq density histograms illustrating 5mC and 5hmC levels at RefSeq genes sorted by RNA-Seq gene expression levels. 5′ and 3′ indicate distal regions and the bar represents the normalized RefSeq transcript length. Color scale: blue depicts lower density with green and red showing higher density.

To explore radiation-regulated methylation dynamics, we generated cytosine methylation (5mC) and/or cytosine hydroxymethylation (5hmC)-DIP-Seq libraries from the hippocampi of mice exposed and not exposed to 1 Gy proton radiation using highly specific antibodies^36, 60^. These libraries were sequenced to a depth of ~40 million tags of which 55–69% mapped to a genomic locus. Genomic regions enriched for 5mC or 5hmC were segmented using a Monte Carlo-based algorithm and the union of these regions was tested for significant difference using a negative binomial approach. This approach identified thousands of differentially methylated and hydroxymethylated regions (DMRs and DHRs, respectively), of which the majority were within 25 kb of a transcription start site (Fig. 5A and B). Consistent with previous studies^61^, 5hmC regions were enriched in intragenic regions and at gene boundaries relative to 5mC (Fig. 5C). Both 5mc and 5hmC regions were enriched at major parasitic repeats (LINEs, SINEs, LTR-based repeated) with 5mC showing the expected enrichment at Satellite repeats (Supplemental Fig. 4). We next generated heatmaps that depict 5mC and 5hmC DIP-Seq density relative to Ref-Seq gene expression levels (Fig. 5D). As expected, the global 5mC signal was depleted at the transcriptional start sites of active genes and enriched intragenically at genes expressed at low levels. In contrast, the 5hmC signal showed marked enrichment at active genes that tailed off as expression decreased. These plots are consistent with our previous reported data^60^ and those of others^62^ and highlight the differential regulatory roles of these two distinct epigenetic marks in hippocampus.

**Figure 6:**
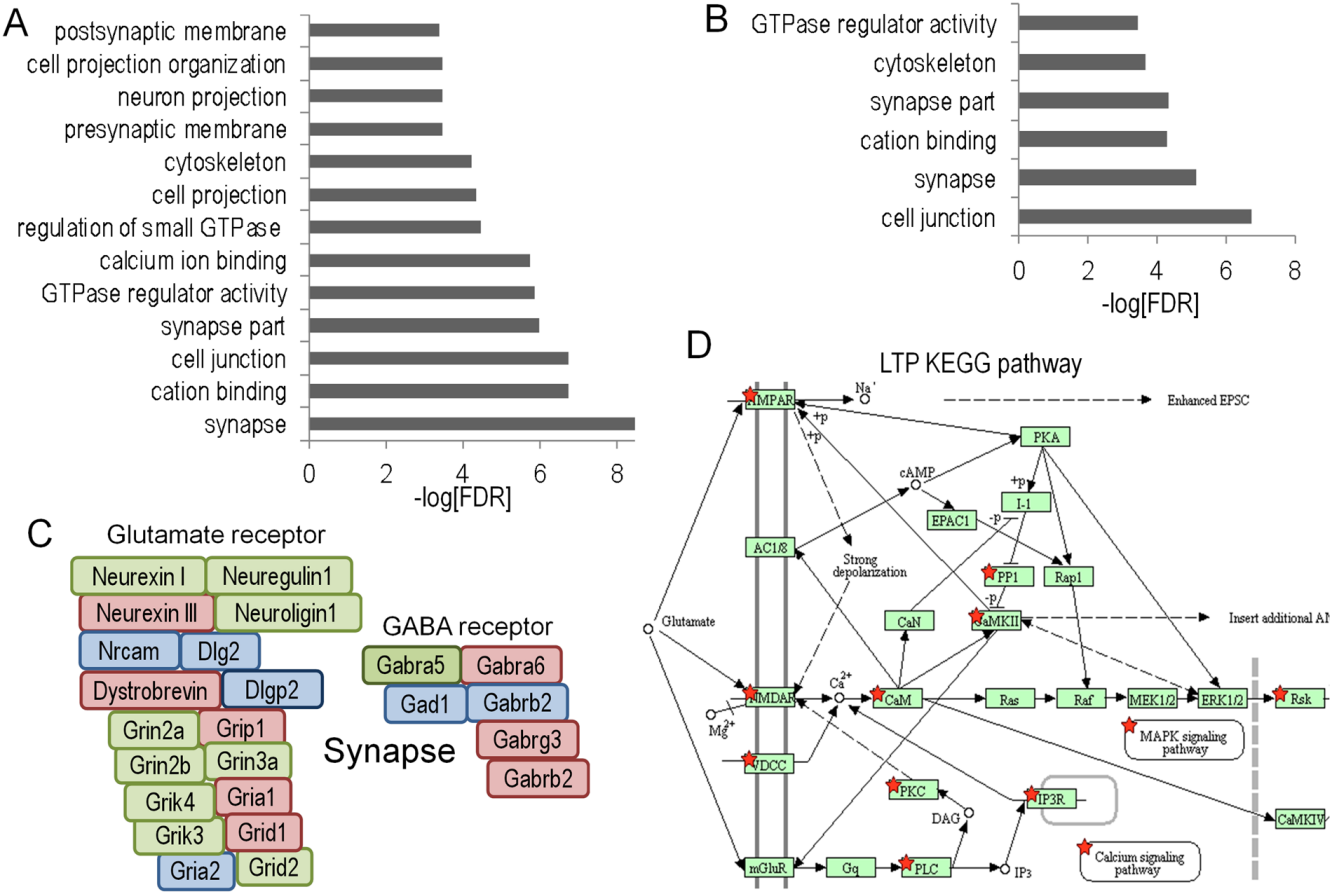
Gene ontology analyses of upregulated 5hmC DHRs (**A**) and downregulated DHRs. (**B**) DHRs were annotated with the closest RefSeq gene start site within 50 kb. (**C**) Selected genes from the synapse gene ontology category. Green depicts genes found in upregulated DHRs, red depicts genes found in down-regulated DHRs and blue depicts genes found in both categories. (**D**) KEGG pathway analysis shows that up and down-regulated DHRs are both enriched for the same set of LTP-associated genes. *p* < 1 × 10^−6^. The KEGG database was developed by the Kanehissa Laboratories, as described^75^.

Gene ontology analysis of significantly-regulated DMR and DHRs identified gene ontology categories for the 5hmC data (Fig. 6A and B), with both increased and decreased levels observed, but not for the 5mC data (not shown). Many of these categories were specific to synaptic function and neuronal function. Examples of genes that are differentially-regulated for 5hmC in the synapse category are illustrated in Fig. 6C, though we note that the observed changes were not limited to a single directional change (*e.g*., all down-regulated). Instead, we observed genes within specific gene ontology pathways with increased or decrease 5hmC levels, or both (see below). These results suggest that dynamic changes in 5hmC after proton exposure are more strongly associated with gene pathways and that changes within these pathways are complex.

**Figure 7:**
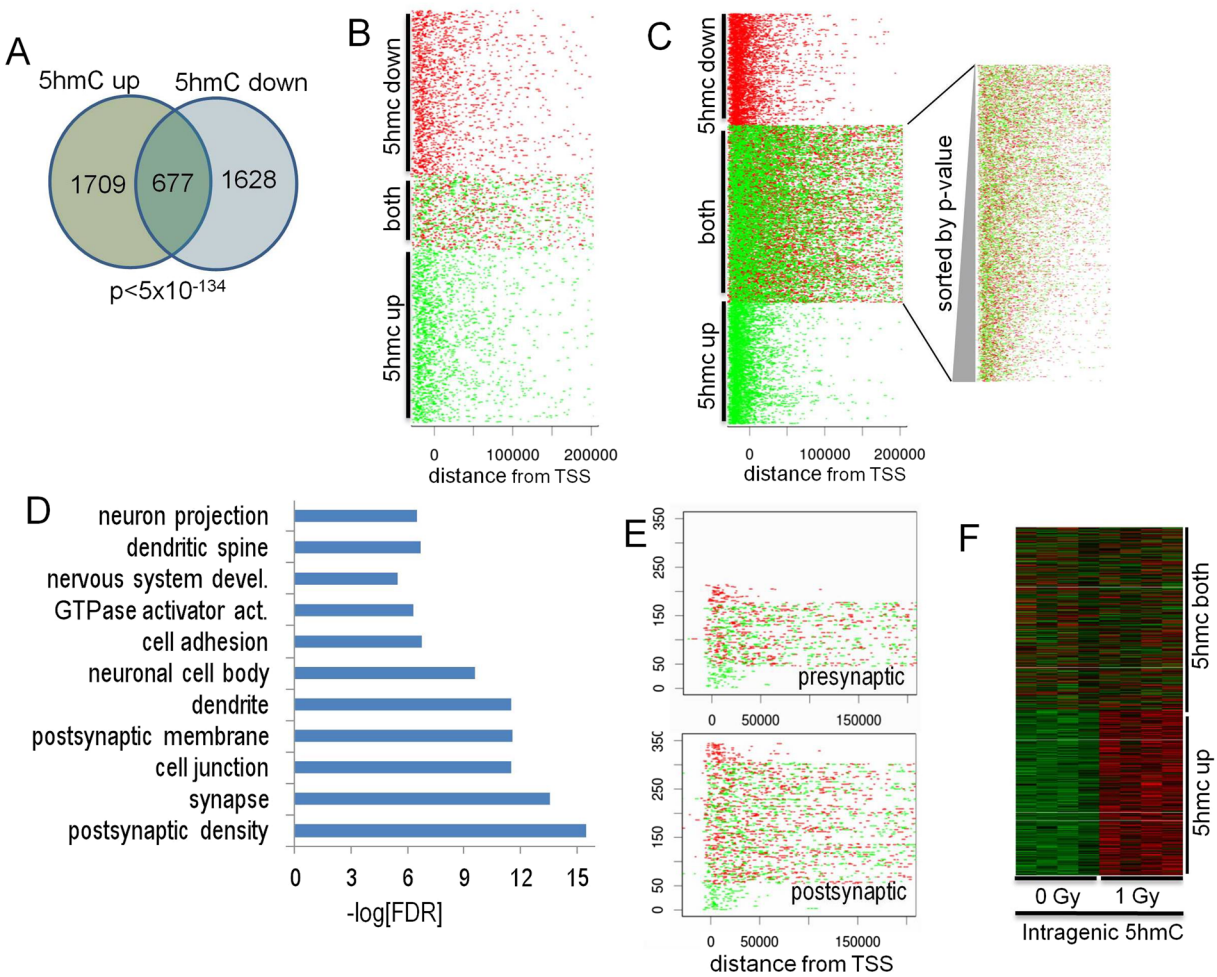
Dynamic directional regulation of 5hmC at the same locus enriches for synaptic pathways. (**A**) The Venn diagram illustrates that a highly significant percentage of genes are associated with both an up- and down-regulated DHR. (**B**) Genes associated with both increased and decreased DHRs had significantly higher intragenic DHR density. (**C**) Analysis of genes with both increased and decreased DHRs revealed that the increased density was highly correlated with DHR significance (ranked by most significant individual DHR). (**D**) GO analyses of the 677 genes associated with bidirectional DHR regulation identified categories associated with synapse and neuronal function. (**E**) Enrichment for bidirectional regulation of DHRs at postsynaptic gene loci. (**F**) The heatmap depicts row-scaled 5hmC sequence-density of the most significant DHRs associated with increased DHR genes and genes with both increased and decreased DHRs. Red denotes higher 5hmC sequence density and green denotes lower density.

KEGG analyses revealed that the long-term potentiation (LTP) (Fig. 6D) and synapse pathways (Supplemental Fig. 5, Supplemental Table 2) were significantly associated with both increased and decreased DHRs. Remarkably, these sets of genes were mostly identical. In other words, regions identified with increased 5hmC levels were also often identified because they exhibited decreased 5hmC levels (Fig. 7A). This high-degree of region-associated overlap, between increased and decreased DHRs, is depicted in a heatmap that represents each up-regulated DHR in green and each down-regulated DHR in red (Fig. 7B). Importantly, the heatmap shows that while these regions are in close proximity they do not overlap. We explored a number of trivial explanations for this phenomenon, including the potential that this overlap is the consequence of differences in 5hmC signal density, but we found no difference in global 5hmC density levels at genes with overlapping up and down DHRs versus up alone or down alone (Supplemental Fig. 6). Moreover, when we examined individual GO categories, we did not find evidence of significant differences in overall 5hmC levels (data not shown and Fig. 7D). Differences in transcription or splicing could also explain bidirectional intragenic regulation of 5hmC. Although intragenic bidirectional 5hmC accumulation was highly correlated with DHR significance, no significant correlation with transcriptional regulation was observed (data not shown). Because an association between methylation and splicing has been previously described^63, 64^, we analyzed proton-radiation differential exon usage (DEU). Only 4 DEU events were detected (FDR-adjusted *p* < 0.01; Supplemental Fig. 7A,B). We compared potential DEU (at a more permissive unadjusted *p* < 0.001) with annotated- DHRs found that they were less likely to be associated with bidirectional DHRs than control “unidirectional” DHRs (Supplemental Fig. 7C). A general association of DHRs with DEU is not surprising, given the documented enrichment for 5hmC in transcriptionally active regions^61, 65^.

Genes associated with DHRs that were both increased and decreased appeared to contain a higher density of DHRs than genes associated with unidirectional changes. To better assess this trend, we examined gene-associated DHR density using a more permissive threshold (*p* < 0.1). Surprisingly, this analysis suggested that genes associated with both increased and decreased DHRs had higher intragenic DHR density and that these changes extended across entire gene bodies, as opposed to unidirectional changes that tend to cluster at the 5′ ends (Fig. 7B and C). To determine whether the apparent increase in DHR density was significant, we normalized for length by removing small genes (<200 kb) and measured DHR density in four 50 kb bins distal to the gene start. The bin near the start site was not significantly different from controls but bi-directional DHR density increased significantly along the direction of transcription (Supplemental Fig. 8). Further analysis of these bi-directionally-affected genes revealed that the increased density was highly correlated with DHR significance (ranked by most significant individual DHR) (Fig. 7C).

GO and KEGG pathway analyses of the 677 genes associated with bidirectional DHR regulation identified categories associated with synapse and neuronal function (Fig. 7D). Interestingly, GO category significance for this subset of genes was increased markedly over the analysis for all DHR regions (Fig. 6A). Moreover, a marked enrichment was observed for postsynaptic GO categories, but not presynaptic categories (Fig. 7C). Importantly, bidirectional DHR density was not significantly different between the postsynaptic and presynaptic GO category (Fig. 7E, *p* = 0.2). Bidirectional regulation of DHRs for individual replicates is shown via heatmaps (Fig. 7F) that depict 5hmC signal at the most significant DHRs for the overlapping genes in Fig. 7A. These data show that proton irradiation can induce both up- and down-regulation of 5hmC at a subset of gene-associated loci in a spatially-restricted manner. Moreover, this novel DHR subset is associated with a marked increase in intragenic DHR density and highly significant enrichment for genes associated with the postsynaptic specialization demonstrating remarkable specificity for the response to proton irradiation.

We next explored whether bidirectional regulation of 5hmC also occurred in the brains of mice irradiated with a markedly different form or ionizing radiation, ^56^Fe ions, that we examined in a prior study^60^. After reanalyzing the iron data using a similar bioinformatics pipeline, we again observed a strikingly significant overlap between increased and decreased DHRs at the same genes for both the 0.1 Gy and 0.2 Gy doses (Supplemental Fig. 8A,B). GO pathways analyses identified highly specific enrichment for genes associated with nervous system development, synapse function, post-synaptic density, and positive regulation of transcription at two doses (0.1 Gy and 0.2 Gy) (Supplemental Fig. 8C,D). The highly significant enrichment for transcription-associated genes was not seen for protons suggesting differences in the radiation response for bi-directionally-regulated DHRs that are consistent with our observation of a greater number of transcriptional changes after^56^ Fe ion exposure^66^.

**Figure 8:**
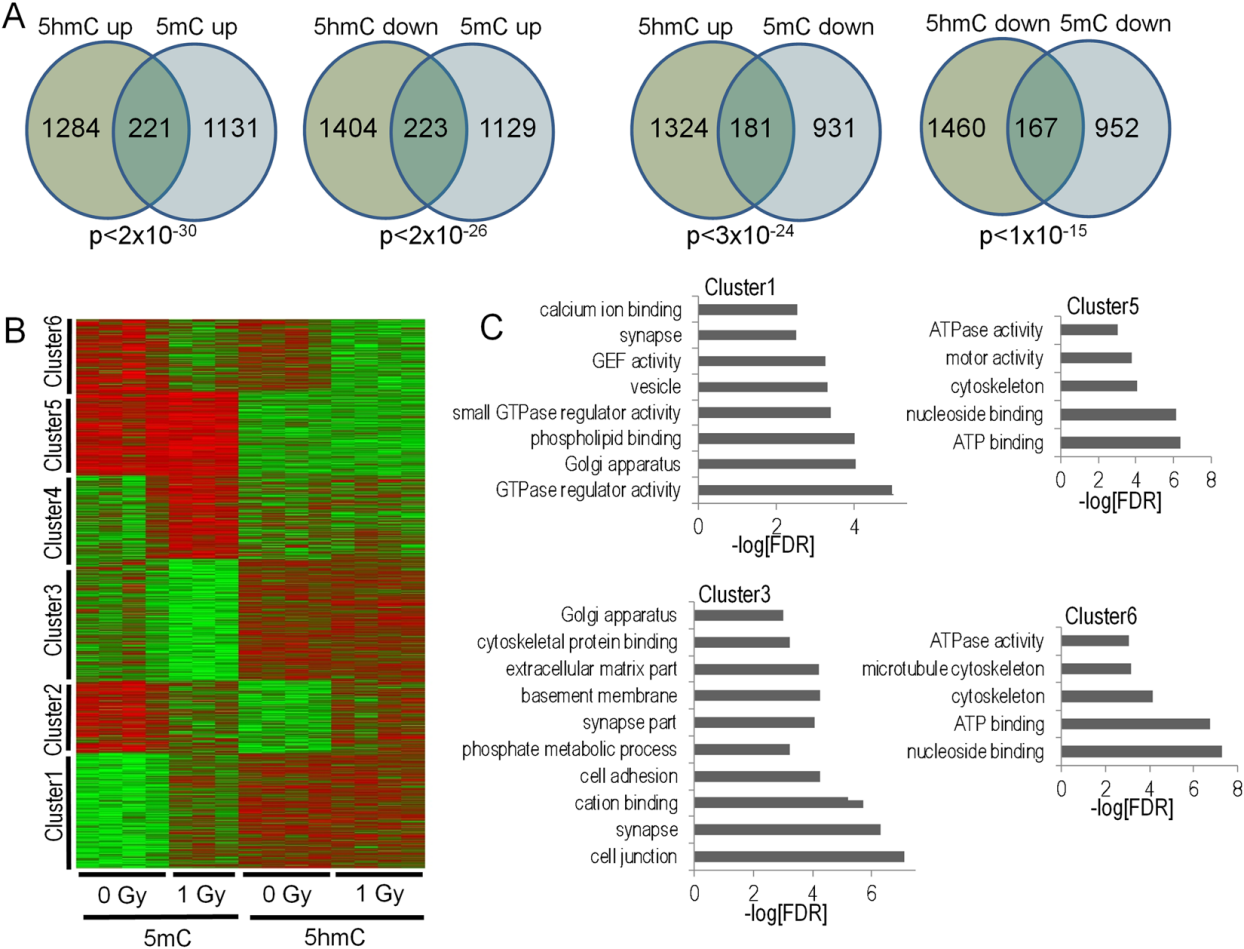
(**A**) Venn diagrams depict the overlaps of gene-associated DMRs based on the direction of change. (**B**) Unsupervised K-means clustering reveals distinct patterns of 5hmC and 5mC regulation. (**C**) Gene ontology analyses of 5mc-5hmC clusters reveals significant enrichment for novel gene pathways (not revealed in the individual analyses of 5mC and 5hmC).

We next examined the relationship between proton-regulated gene-associated DMRs and DHRs. Consistent with previous studies we found significant overlap irrespective of the direction of change (Fig. 8A). The most significant overlap was found for genes that were associated with increases in both 5mC and 5hmC. This result is consistent with the dependency of 5hmC for pre-existing or concomitant DNA methylation, as is the group in which 5mC goes down and 5hmC goes up. To explore these relationships in greater detail we performed unsupervised K-means clustering of gene-associated DMRs and DHRs (Fig. 8B). Interestingly, this analysis identified regulatory clusters associated with gene ontology categories that differed markedly from our global gene ontology analyses in Fig. 6, again demonstrating specificity for the response to irradiation. For example, Cluster 3 showed a decrease in proton radiation-induced 5mC, but overall higher-levels of 5hmC and was enriched for synapse-associated categories while Cluster 1 showed an increase in proton radiation-induced 5mC levels, but overall higher levels of 5hmC, and was enriched for genes linked to G protein function and intracellular signaling.

This clustering analysis also shed light onto the question of whether changes in 5hmC depend on pre-existing 5mC or coordinated deposition of 5mC. Consistent with the idea that 5hmC depends on 5mC, we did not identify clusters that showed a change in 5hmC without a corresponding change in 5mC. Intriguingly, we also did not identify a cluster where radiation-induced increases in 5mC were associated with changes in 5hmC. This result suggests that radiation-induced changes in 5hmC predominantly utilize pre-existing pools of 5mC.

### The relation between gene expression and DNA methylation changes at the two-week post irradiation time point

**Figure 9:**
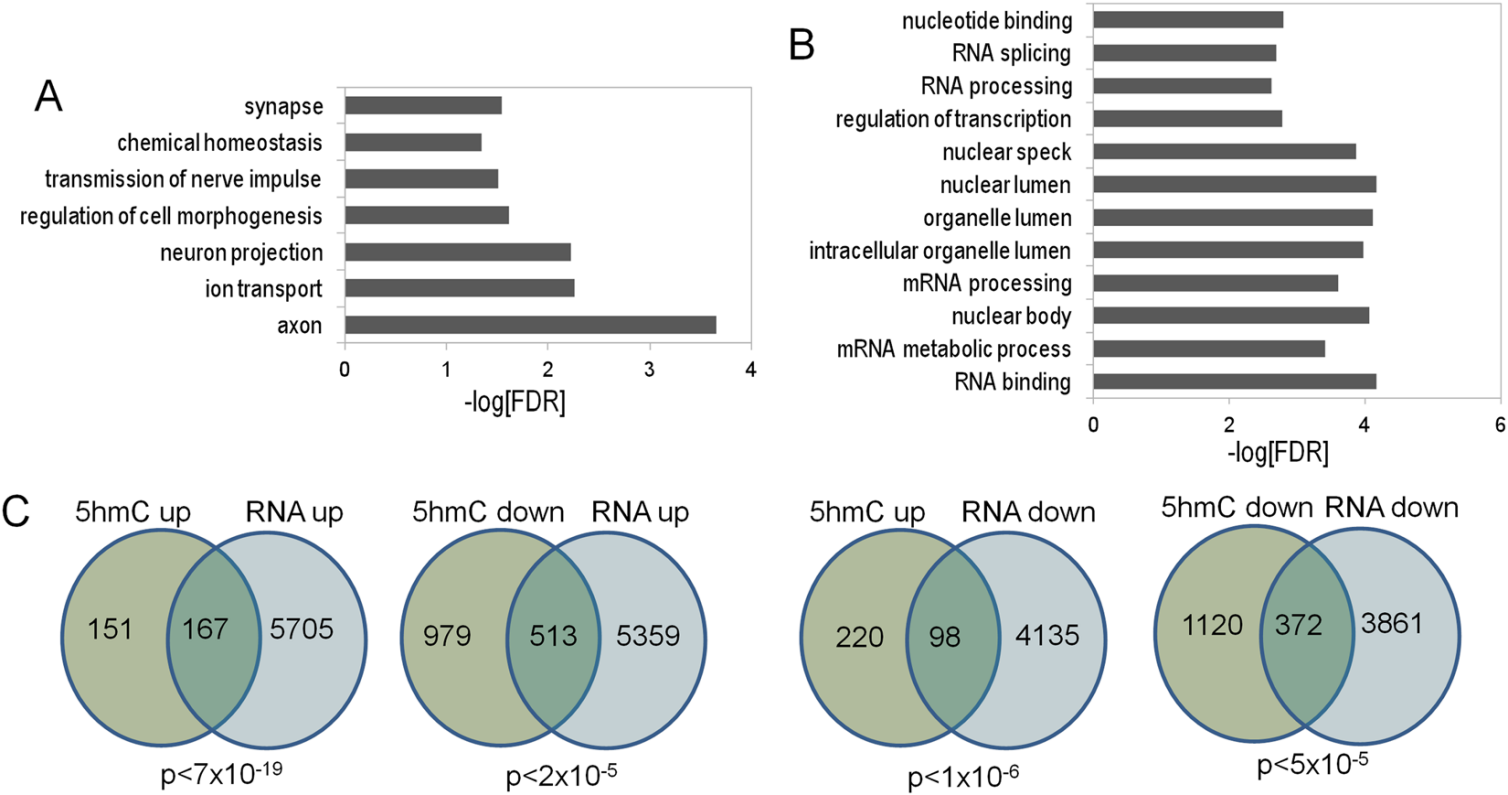
(**A**,**B**) Bar graph depicts gene ontology analyses of RefSeq genes significantly up- and down-regulated at *p* < 0.01. (**C**) Venn diagrams depict the overlap between gene-associated 5hmC DMRs and significantly-regulated genes based on the direction of change.

To examine whether changes in 5mC and 5hmC correlate with changes in transcription, we performed RNA-Seq analyses on RNA-extracted from the same samples removed for the two-week post exposure time point. Although differential expression analyses revealed changes that were relatively modest (Supplemental Table 3), gene ontology analyses of significantly up-regulated genes identified many of the same categories that were previously identified in our analyses of 5hmC (Fig. 9A). In contrast, significantly down-regulated genes revealed categories associated with RNA function and cellular morphology that were not detected in our 5hmC data (Fig. 9B). We next explored whether changes in RNA levels correlated with changes in gene-associated 5hmC. Highly significant overlap was only seen at genes where both 5hmC and gene expression were increased by radiation exposure demonstrating a clear link between increased 5hmC levels and RNA expression at discrete regions of the genome after proton exposure (Fig. 9C).

### Tet2 immunohistochemistry at the two-week time point

We analyzed immunoreactivity of TET2, one of the three TET enzymes responsible for converting 5mC to 5hmC^39^, and likely the most important for brain function^40, 41, 67, 68^, in the hippocampus and cortex at the two-week time point. Tet2 immunoreactivity in the CA1 and CA3 regions of the hippocampus, dentate gyrus, and cortex were comparable in sham-irradiated and irradiated mice (Suppl. Figure 9).

### Effects of proton irradiation on cognitive performance at the twenty-week time point

**Figure 10:**
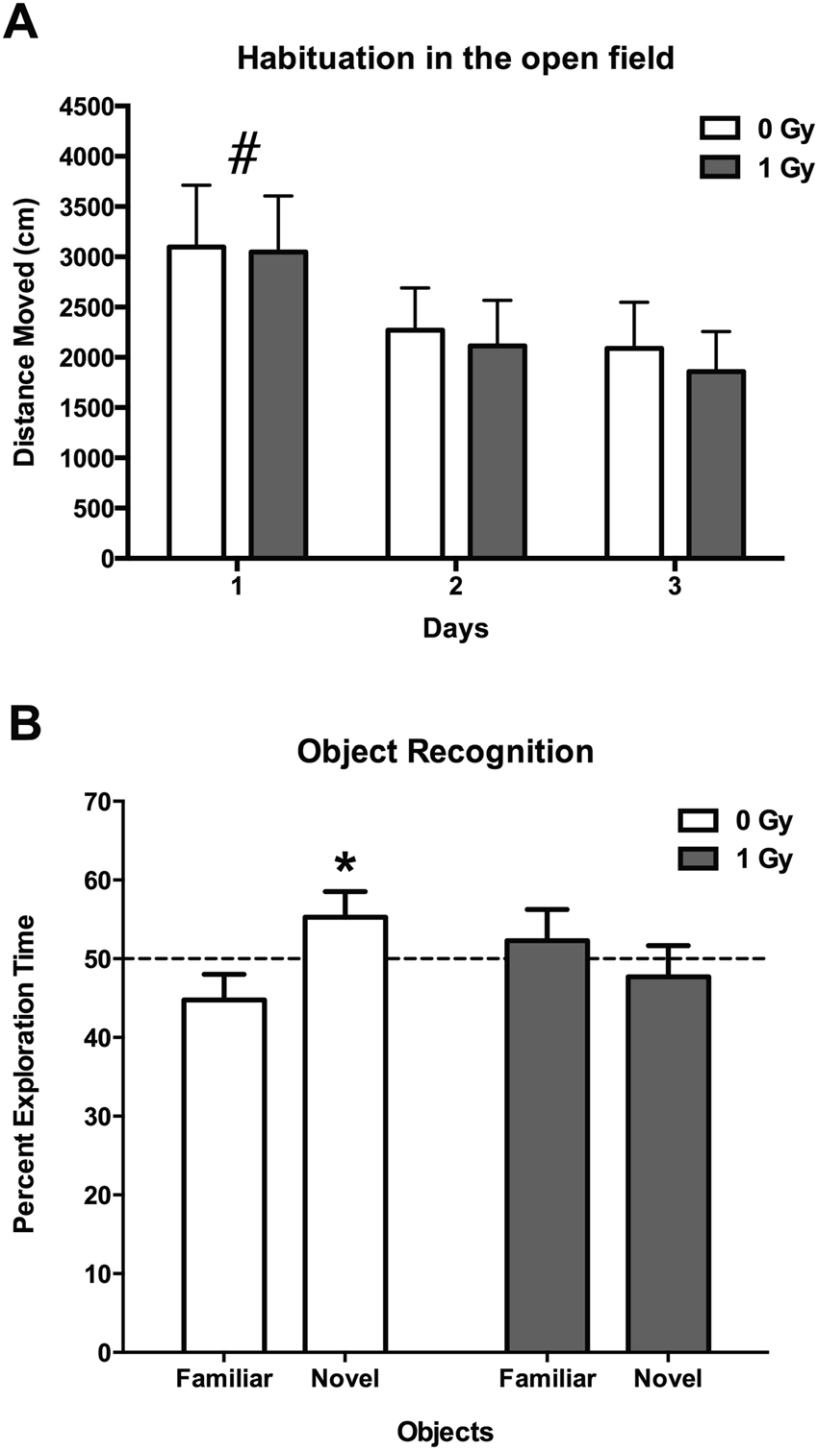
(**A**) Habituation of sham-irradiated and proton-irradiated mice at the 20-week time point. There was an effect of day (*F* = 75.22, *p* < 0.0001), indicating that both groups habituated to the open field. ^#^ *p* < 0.0001 versus days 2 and 3. (**B**) Object recognition at the 2-week time point. Sham-irradiated showed object recognition and spent significantly more time exploring the novel than the familiar object. In contrast, irradiated mice did not. *N* = 16 mice/dose. *p < 0.05.

When activity levels in the open field over three days were analyzed at 20 weeks after exposure, sham-irradiated and irradiated mice habituated to the open field and showed higher activity levels in the open field on the first day than on subsequent days (*F* = 75.22, *p* < 0.0001; Fig. 10A). Significantly, the detrimental effects of proton irradiated on object recognition observed at the 2-week time point was also observed at the 20-week time point. While sham-irradiated mice showed object recognition and spent significantly more time exploring the novel than the familiar object (*t* = 2.267, *p* < 0.05), irradiated mice did not (Fig. 10B).

**Figure 11:**
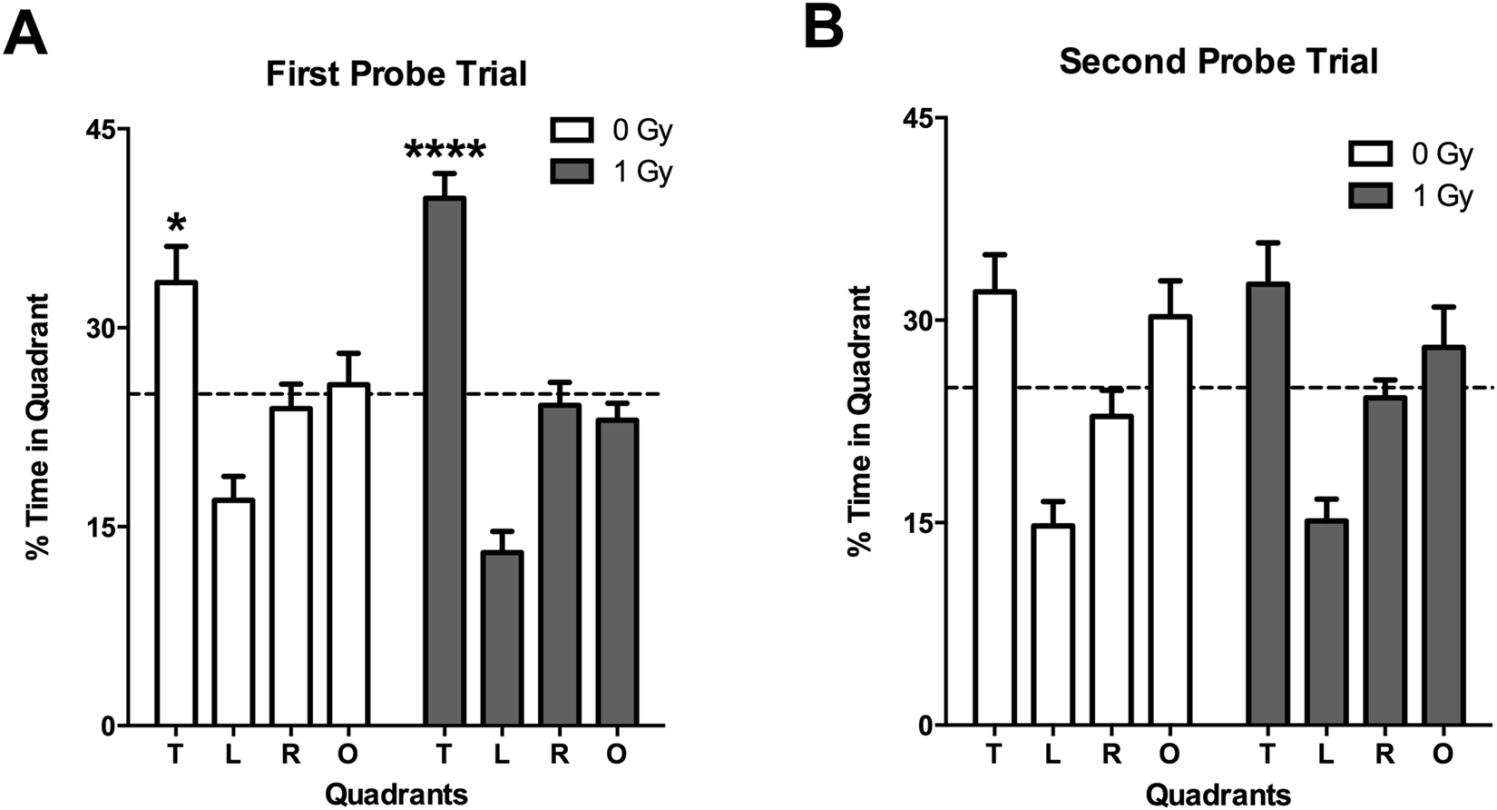
(**A**) Performance in first water maze probe trial at the 20-week time point. Sham-irradiated (effect of quadrant: *F* = 5.605, *p* = 0.01) and proton irradiated mice (effect of quadrant: *F* = 34.3, *p* < 0.0001) spent more time in the target quadrant than any other quadrant. (**B**) Performance in second water maze probe trial at the 20-week time point. Neither group showed spatial memory retention. *N* *=* 16 mice/dose. *Target versus opposite quadrant: *p* < 0.05; Target versus right quadrant: *p* < 0.01; Target versus left quadrant: *p* < 0.001; ***Target versus any other quadrant: *p* < 0.001.

With training, both groups improved their ability to locate a visible and subsequent hidden platform in the water maze (effects of session: *F* = 9.41, *p* < 0.0001) but there was no effect of radiation (not shown). Both groups showed spatial memory retention in the first probe trial, spending more time in the target platform than any other platform (Fig. 11A). This result indicates recovery relative to the 2-week time point. However, neither the irradiated or control group showed spatial memory retention in the second probe trial (Fig. 11B) indicating that long-term memory was impaired in these middle-aged mice, regardless of whether they were irradiated with protons or sham-irradiated.

### Effects of proton irradiation on DNA methylation in the hippocampus at the twenty-week time point

**Figure 12:**
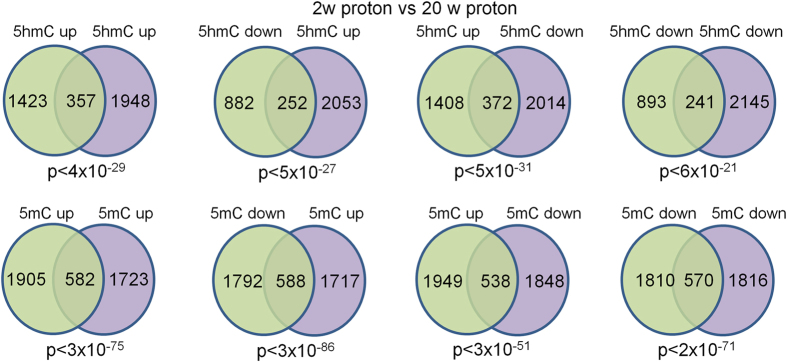
Venn diagrams depicting highly significant overlap between 2-week and 20-week proton DIP-Seq data. DHRs and DMRs were annotated with the closest RefSeq gene start site within 50 kb.

We next sought to compare protonradiation-induced changes in 5mC and 5hmC at the 2-week time point with our previous publication showing changes at the 20-week time point. Because this earlier study used pooled samples we could not directly compare our data. Therefore, we re-analyzed our current data by pooling replicates *in silico* and utilizing the same statistical approach as in the earlier study. This analysis revealed a high-degree of correlation between DMR and DHRs at the 2-week and 20-week time points irrespective of the direction of change (Fig. 12). The overlap was more significant for 5mC suggesting that radiation-induced changes in this mark are more persistent than for 5hmC. Regardless, the highly significant retention of DNA methylation changes from 2-weeks to 20-weeks for both 5hmC and 5mC demonstrates that many of these radiation-induced changes are both specific and stable.

### Tet2 immunoreactivity and the 20-week time point

**Figure 13:**
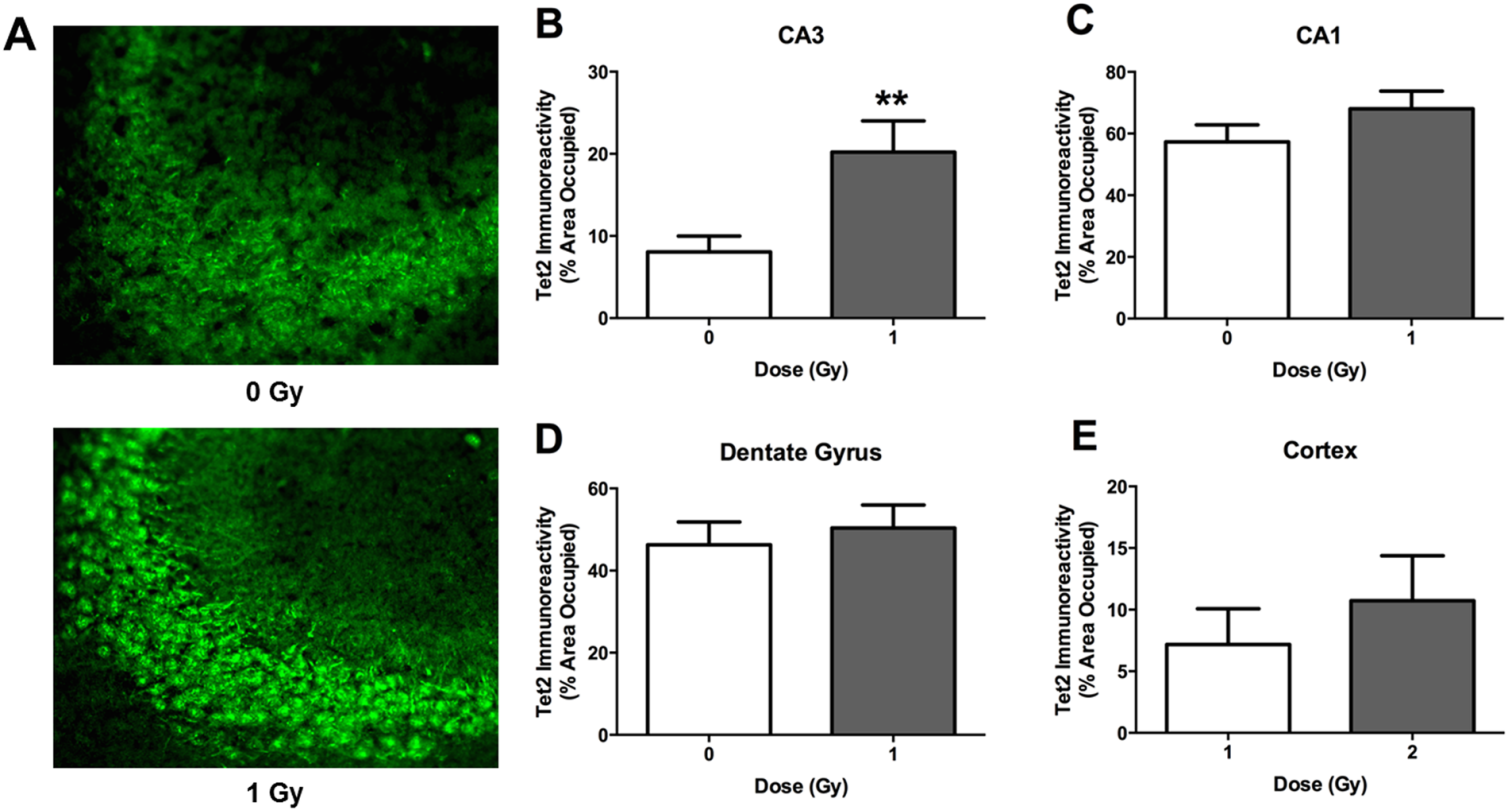
Tet2 immunoreactivity in sham-irradiated and proton irradiated mice at the 20-week time point. (**A**) Representative images of Tet2 immunoreactivity in the CA3 region of the hippocampus of a sham-irradiated mouse and a proton-irradiated mouse. (**B**) Tet2 immunoreactivity levels in the CA3 region of the hippocampus. (**C**) Tet2 immunoreactivity levels in the CA1. (**D**) Tet2 immunoreactivity levels in the dentate gyrus. (**E**) Tet2 immunoreactvity in the cortex. *p < 0.05 versus sham-irradiation. *N* = 19–20 mice/dose.

At the 20-week time point, Tet2 immunoreactivity was significantly higher in the CA3 region of the hippocampus of irradiated mice compared with sham-irradiated mice (*p* = 0.0079, Fig. 13A). In contrast, there were no significant differences in TET2 immunoreactivity in the CA1 region of the hippocampus, dentate gyrus, or cortex of sham-irradiated and irradiated mice (Fig. 13B–D).

### Proton and Fe ion exposures induce overlapping DNA methylation changes

**Figure 14:**
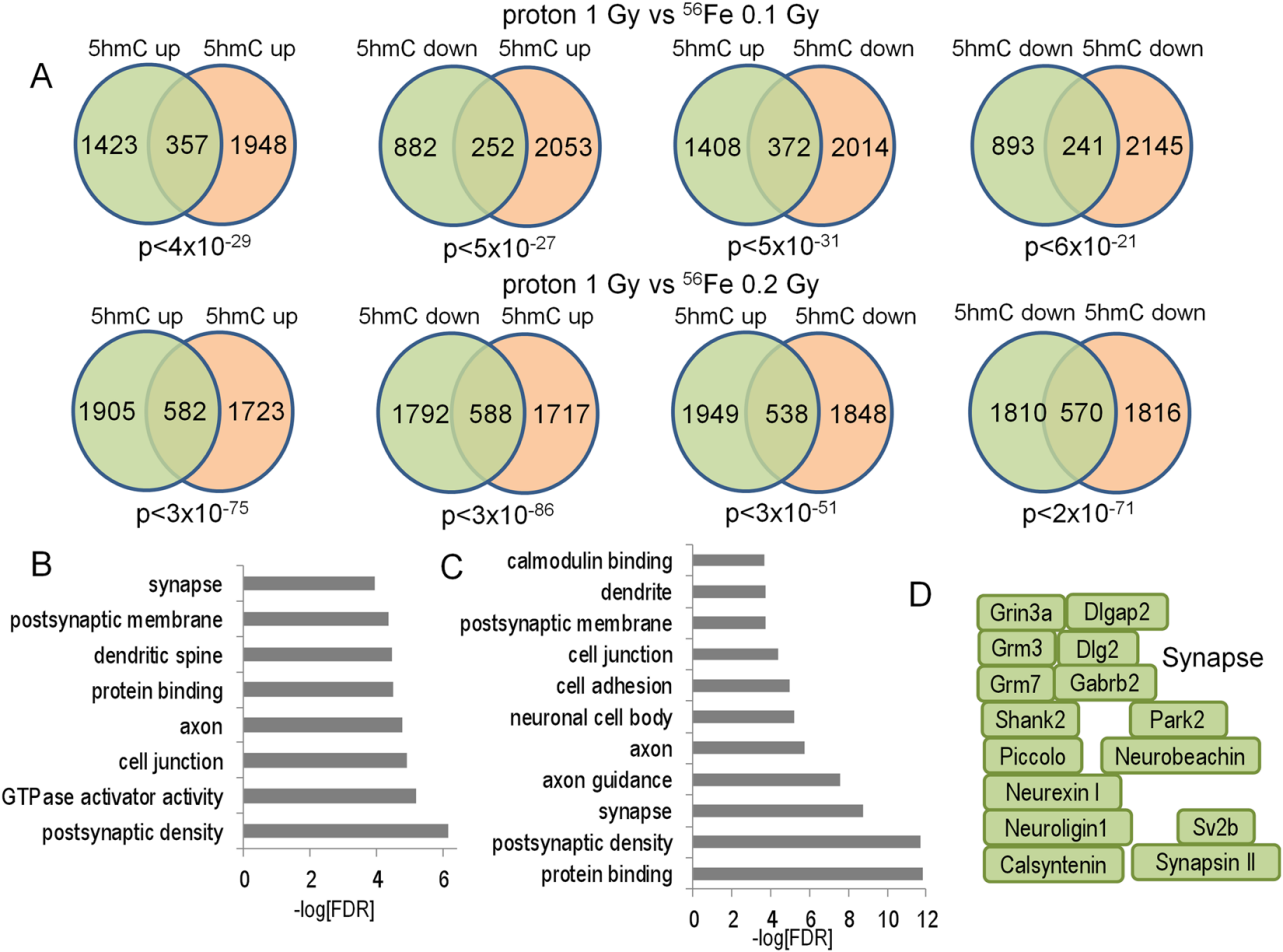
(**A**) Venn diagrams depict overlap between 2-week proton and indicated ^56^Fe DHRs. (**B**) Bar graph depicts gene ontology analyses of 5hmC up-5hmC up DHRs for 1 Gy protons vs 0.1 Gy ^56^Fe. (**C**) Bar graph depicts gene ontology analyses of 5hmC up-5hmC up DHRs for 1 Gy protons vs 0.2 Gy ^56^Fe. (**D**) Examples of genes found in the synapse gene ontology category shown in C.

**Figure 15:**
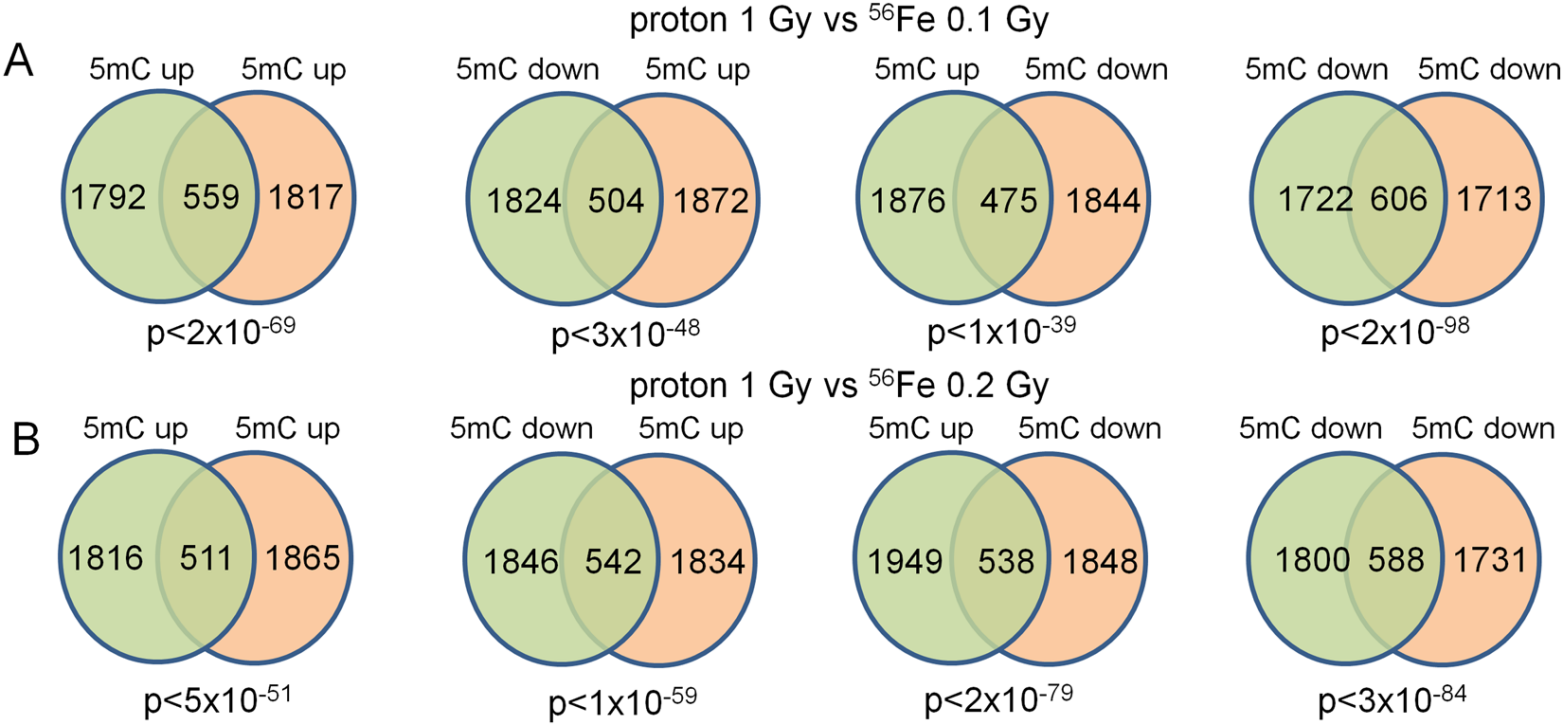
(**A**) Venn diagrams depict overlap between 2-week proton and 2-week^56^Fe 0.1 Gy (**A**) and 0.2 Gy (**B**) DMRs.

To determine if different forms of ionizing radiation induce similar types of DNA methylation changes we compared proton-induced changes in 5mC and 5hmC with those we reported previously for ^56^Fe ion-induced changes.^56^Fe ions are a form of high linear energy transfer (LET) radiation, whereas protons are a form of low LET radiation. Accordingly, we re-analyzed our previously published DIP-Seq data that examined the effects of 0.1 Gy and 0.2 Gy ^56^Fe (600 MeV/n) on hippocampal 5mC and 5hmC at the 2- week time point, which unlike the proton-induced changes partially disappeared at the 20-week time point. Both 0.1 and 0.2 Gy ^56^Fe gene-associated DHRs and DMRs showed a highly-significant correlation with proton DHRs and DMRs from this study (Figs 14 and 15), demonstrating that indeed many of these changes represent a common response to ionizing radiation exposure regardless of its form. Once again, this correlation was highly significant irrespective of the directional change of the DMRs and DHRs. 0.2 Gy ^56^Fe DHRs showed a higher-degree of correlation with the proton data and gene ontology analyses of the overlapping iron and proton DHRs showed a high-degree of concordance with the neuronal categories described for proton DHRs in this manuscript (Fig. 14B–D, Supplemental Table 4). Gene ontology analyses of the overlapping iron and proton DMRs revealed categories linked to cell adhesion, cell junctions, neuronal growth, and synapse function (Supplemental Table 4).

## Discussion

In this study, we examined the short- and long-term effects of whole body proton irradiation on hippocampus-dependent cognitive function, hippocampal DNA methylation, and hippocampal RNA expression. Our data demonstrate a clear relationship between changed gene expression and one form of DNA methylation (5hmC) after exposure and suggest altered gene ontology and KEGG pathways that account for altered cognitive function. Cognitive changes occur relatively rapidly because impairment for object recognition, spatial memory retention in the water maze, and network stability following proton irradiation were observed at the two-week time point. Our data also showed epigenetic remodeling of a subset of genes with critical neuronal functions within four weeks of exposure.

The DNA methylation data for the unexposed hippocampus showed anticipated relationships between 5mC and gene expression, *i.e*., depletion at the transcriptional start sites of active genes and enrichment at genes expressed at low levels, and 5hmC and gene expression, *i.e*., high levels for active genes and low levels for inactive genes. These data show differential biological function for these two forms of DNA methylation, which did not respond identically to proton exposure. Changes in 5hmC levels were more likely to localize to genes required for synaptic and neuronal functions, suggesting that dynamic changes in 5hmC are more strongly associated with functionally significant gene pathways. Strikingly, a significant percentage of genes exhibited both increased and decreased intragenic 5hmC levels, suggesting that proton irradiation triggers a complex remodeling at some gene-associated loci in a spatially-restricted manner. In contrast, 5hmC changes at genes exhibiting only increased or decreased levels were restricted more to their 5′ ends. Because bi-directionally-affected DHR density increased in significance distal to transcriptional start sites, it is conceivable that 5hmC remodeling is linked to transcription or RNA processing. Interestingly, a recent study found an association between dynamic regulation of 5hmC in response to cocaine administration and alternative splicing^66^. It would be interesting to determine whether transcripts enriched for bidirectional DHR remodeling are associated with changes in RNA transcription or splicing and whether bidirectional and unidirectional 5hmC remodeling generalizes to other experimental models. These results suggest that DNA methylation changes as a result of proton exposure can occur via multiple pathways and that these pathways are gene specific.

When we analyzed changes in 5hmC that correlate with changes in transcription, significant overlap was observed for genes within neuronal gene ontology pathway that exhibited both increased 5hmC levels and transcription demonstrating a clear relationship, but whether one change precedes the other or if they occur simultaneously is not known. A kinetic analysis will be necessary to address this question. In addition, the RNA-seq data identified other gene ontology pathways associated with RNA function and cellular morphology that were not detected in our 5hmC analysis.

Consistent with the gene ontology analyses, KEGG pathways linked to synaptic function and neuronal processes were significantly enriched for gene-associated DHRs. For example, the long-term potentiation pathway was enriched for both up- and down-regulated DHRs. The genes present in the up and down-regulated long-term potentiation KEGG category showed perfect overlap, highlighting the dynamic and spatially-restricted regulation of 5hmC at gene loci by proton radiation. The data suggest that radiation-induced changes in 5hmC may predominantly utilize pre-existing pools of methylation. Genes that are expressed at higher levels in the hippocampus were not more affected than those expressed at lower levels and the variance in 5hmC was similar in the sham- and proton-irradiated mice.

At the 20-week time point, object recognition, but not spatial memory retention in the probe trial following the first day of hidden platform training, was impaired in proton irradiated mice. In addition, TET2 levels in the CA3 region of the hippocampus were increased following proton irradiation at this time point. Comparing proton-radiation-induced changes in 5mC and 5hmC at the 2-week and 20-week time points revealed a high-degree of correlation between DMR and DHRs at the 2-week and 20-week time points irrespective of the direction of change. The overlap was far more significant for 5mC demonstrating that radiation-induced changes in this mark are more persistent than for 5hmC, despite the lack of correlation between 5mC and RNA expression changes. Despite this lack of correlation, we showed in a prior study that these 5mC changes are tissue specific^36^, which demonstrates functional significance for these stable changes that relate to tissue specific functions. Further work will be required to determine the functional significance of these stable 5mC changes that result from proton exposure. The DNA methylation data are consistent with involvement of post-synaptic rather than pre-synaptic mechanisms in the effects of proton irradiation. A striking observation was the enrichment for 5hmC changes near NMDA-subtype glutamate receptors and genes linked to LTP, both of which have been linked to alterations in postsynaptic function. Interestingly, changes in NMDA receptor composition in response to radiation have been observed in rodent models and in human patients^69, 70^ Consistent with this, following proton irradiation increases in hippocampal levels of postsynaptic density protein 95 (PSD95)^71^ and an increase in the rate of miniature excitatory postsynaptic currents of in the CA1 hippocampal region were reported^72^. Genes that are differentially-regulated in the synapse category at the 2-week time point include the GABA receptor (Fig. 5E). Interestingly, following 5–9 weeks after proton irradiation (150 MeV, 0.5 Gy) of 2–3 month-old C57BL/6 J mice, there was increased GABA release from the cannabinoid type 1 receptor (CB1)-expressing basket cells (CB1 BCs) onto pyramidal cells^73^.

In a prior study, we examined the effects of accelerated^56^Fe ion irradiation of DNA methylation in the hippocampus^60^. When we analyzed whether proton radiation-induced changes in 5mC and 5hmC correlate with those seen for ^56^Fe ion irradiation-induced changes, both 0.1 and 0.2 Gy ^56^Fe gene-associated DHRs and DMRs showed a high-significant correlation with proton DHRs and DMRs, irrespective of the directional change, and this correlation was stronger for 0.2 than 0.1 Gy ^56^Fe ions, consistent with our observation that DNA methylation changes were greater at this dose. Gene ontology analyses of the overlapping ^56^Fe and proton DHRs had a high-degree of concordance with the neuronal categories described for proton DHRs, revealing categories linked to cell adhesion, cell junctions, neuronal growth, and synapse function. Our comparison of protons and ^56^Fe ions also showed that changes in distinct pathways, demonstrating a radiation quality effect. Thus, while the detrimental effects on cognitive function and network stability-related outcome measures might be the same, the pathways responsible for these changes after exposure to different particles might be very different. This is especially a concern for NASA in the context of planned long-term space missions, such as those to Mars, because astronauts will be exposed to different radiation qualities during a space mission. Additive or even synergistic effects on pathway changes that result in more profound and longer-term effects on the brain might occur. These data also highlight the need to include mixed space radiation exposures in the experimental paradigm^74^.

The short- and long-term effects of proton irradiation on DNA methylation could not be accounted for by radiation-induced changes in TET2 protein levels. The TET2 protein levels were only significantly increased in the CA3 region of the hippocampus at the 20-week time point but no difference in TET2 protein levels in any brain region analyzed was seen at the 2-week time point. Interestingly, this pattern was also distinct from that seen 2 and 20 weeks following ^56^Fe ion irradiation, further supporting the involvement of different pathways following proton and ^56^Fe ion irradiation.

The distinct hippocampal outcome measures (object recognition and spatial memory retention in the water maze) used in this study showed similar susceptibility to detrimental effects of proton irradiation at the 2-week time point but differential susceptibility to these effects at the 20-week time point with impaired object recognition but not impaired spatial memory retention. These data indicate that the object recognition test might be particularly sensitive to detect detrimental effects of proton irradiation. Impaired object recognition was seen three months, but not one month, following proton (150 MeV, 0.1 Gy) only or mixed proton and ^56^Fe irradiation of 2–3 month-old C57BL/6 J mice^27^. Future studies are warranted to compare the effects of 0.1 and 1 Gy proton irradiation in 2–3 month-old and 6-month-old mice at 1 month after exposure to determine the role of the age of the animal at the time of irradiation on cognitive performance.

It is intriguing that the pattern for the total number of *Arc*-positive cells and the percentage of neurons expressing *Arc* in the nucleus and cytoplasm was similar in sham-irradiated mice exposed to the same environment and proton-irradiated mice exposed to different environments. This pattern, showing a mirror image of opposing effects of environment in the sham-irradiated and irradiated groups, might be specific to proton irradiation as we did not see this following ^56^Fe ion irradiation^55^. There seem two impairments here: 1) inability for proton- and ^56^Fe ion-irradiated mice to remember that they were in the same environment before. So, that would explain why following exposure to the same environment there the *Arc* outcome measures are lower in irradiated than sham-irradiated mice; and 2) inability to detect a novel environment and, as a result, an environment that is clearly different, the proton-irradiated mice experience as identical and therefore they show the same pattern as seen in sham-irradiated mice that are exposed to different environments. So, the proton-irradiated mice must pick up some clues as to what environment they are in but cannot detect the difference.”

In summary, proton irradiation has short- and long-term detrimental effects on object recognition and is associated with short- and long-term alterations in 5hmC in the hippocampus. There is a fundamental epigenetic remodeling for a subset of genes with critical neuronal functions and other cellular functions as a result of exposure. The long-term detrimental effects of proton irradiation on hippocampal function are remarkable, considering that the effects of ^56^Fe ion irradiation were more pronounced at 2 rather than 20 weeks following exposure. Post-synaptic synapse remodeling might be particularly important in these effects. Comparison of changes in DNA methylation following irradiation with protons and ^56^Fe showed an overlap in changes in distinct pathways, demonstrating a radiation signature. Future efforts are warranted to compare these distinct effects of proton and ^56^Fe ion irradiation on hippocampal function with those of another heavy ion exposure such as ^28^Si ion exposure, how these radiation quality-dependent changes might interact, and finally how they occur.

## Electronic supplementary material

Suppl file

Suppl Table 1

Suppl Table 2

Suppl Table 3

Suppl Table 4
