# Supplementary material for: Bi-directional and shared epigenomic signatures following proton and 56Fe irradiation
Source: Sci Rep. 2017 Aug 31;7:10227. doi: 10.1038/s41598-017-09191-4 (PMC5579159; doi:10.1038/s41598-017-09191-4)
Supplement: Supplementary file 1 — Suppl file [file 41598_2017_9191_MOESM1_ESM.pdf]

Bi-directional and shared epigenomic signatures following proton and ^{56}Fe irradiation

Running title: Proton irradiation, cognition, DNA methylation, and gene expression

Soren Impey [a, b #], Timothy Jopson [c], Carl Pelz [a], Amanuel Tafessu [a], Fatema Fareh [a], Damian Zuloaga [d], Tessa Marzulla [d], Lara-Kirstie Riparip [c], Blair Stewart [d], Susanna Rosi [c], Mitchell S. Turker [e], and Jacob Raber [d, f #]

[a] Oregon Stem Cell Center and Department of Pediatrics, Oregon Health and Science University, Portland, OR 97239 USA

[b] Department of Cell and Developmental Biology, Oregon Health and Science University, Portland, OR 97239 USA

[c] Brain and Spinal Injury Center, Departments of Neurological Surgery and Physical Therapy and Rehabilitation Science, University of California, San Francisco, San Francisco, CA, 94110 USA

[d] Department of Behavioral Neuroscience, Oregon Health and Science University, Portland, OR 97239 USA

[e] Oregon Institute of Occupational Health Sciences and Department of Molecular and Medical Genetics, Oregon Health and Science University, Portland, OR 97239

[f] Departments of Neurology and Radiation Medicine, Division of Neuroscience ONPRC, Oregon Health and Science University, Portland, OR 97239, USA

Proton 2w 0 Gy

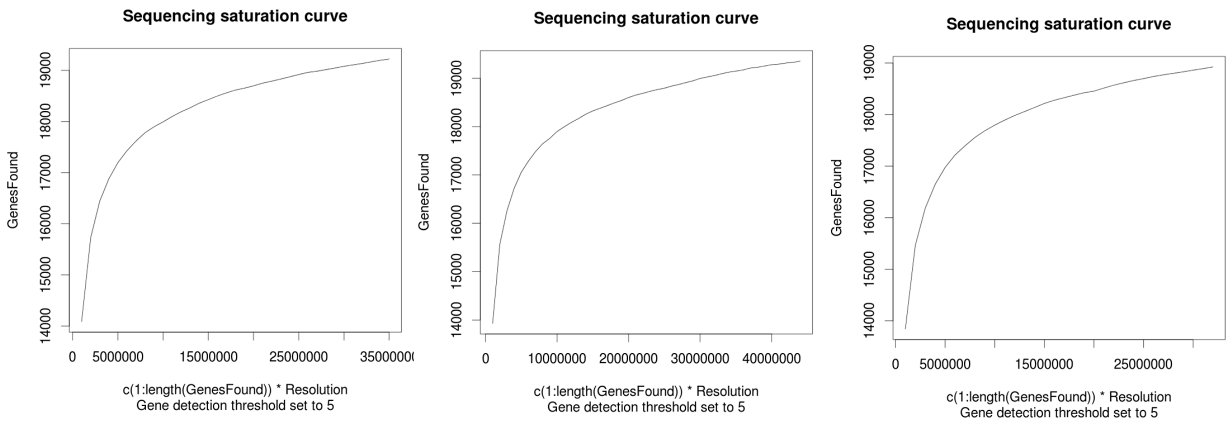

Proton 2w 1 Gy

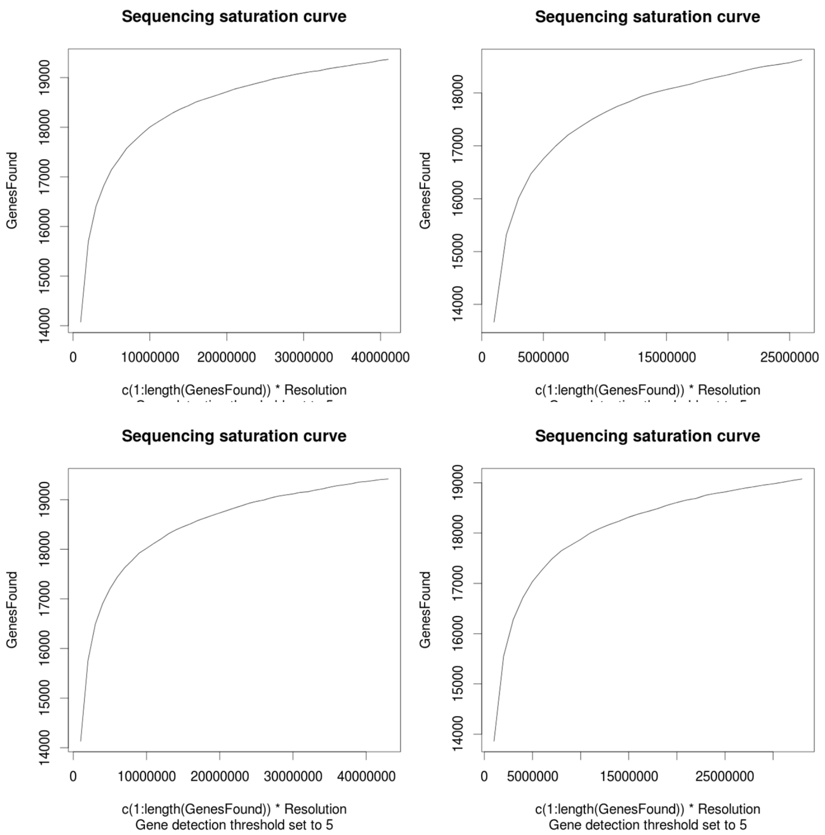

Suppl Fig 1

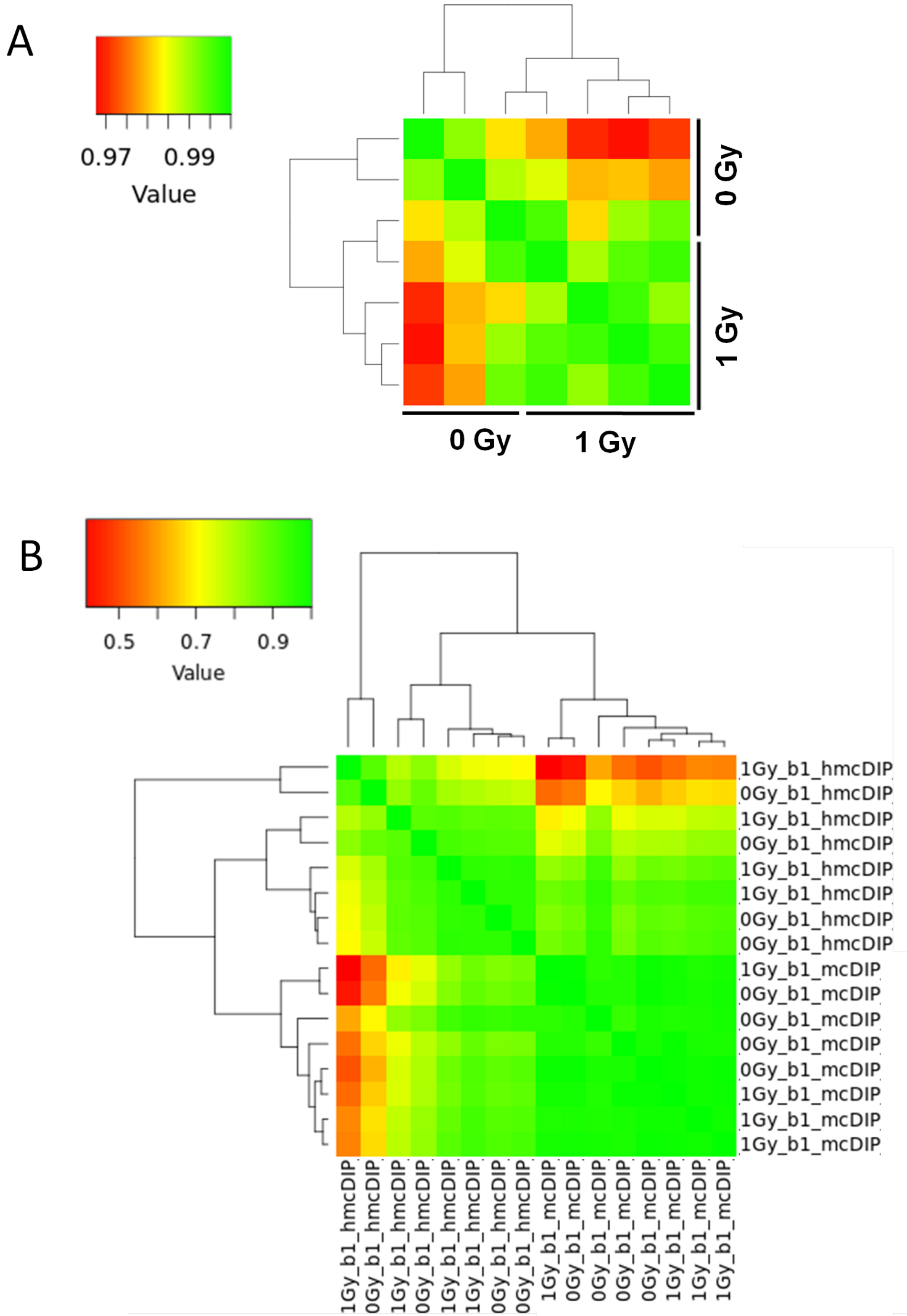

A
0.97
0.99
Value
0 Gy
1 Gy
0 Gy
1 Gy
B
0.5
0.7
0.9
Value
1Gy_b1_hmcDIP
0Gy_b1_hmcDIP
1Gy_b1_hmcDIP
0Gy_b1_hmcDIP
1Gy_b1_hmcDIP
1Gy_b1_hmcDIP
0Gy_b1_hmcDIP
0Gy_b1_hmcDIP
1Gy_b1_mcDIP
0Gy_b1_mcDIP
0Gy_b1_mcDIP
0Gy_b1_mcDIP
0Gy_b1_mcDIP
1Gy_b1_mcDIP
1Gy_b1_mcDIP
1Gy_b1_mcDIP

Suppl Fig 2

Scatter Plot 5mC vs 5hmC

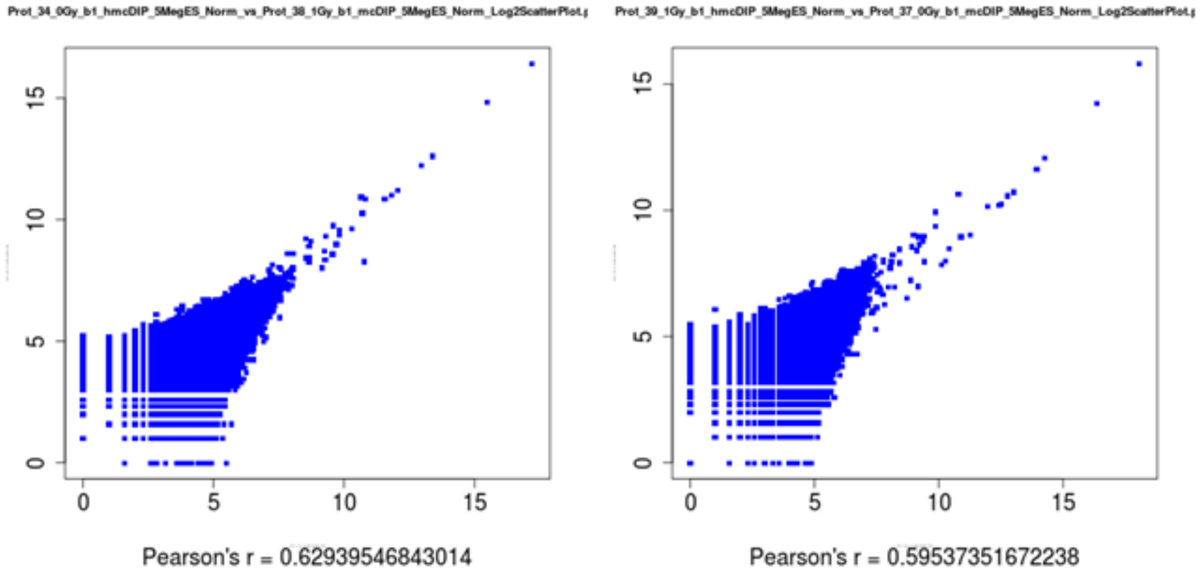

Scatter Plot 5hmC vs 5hmC

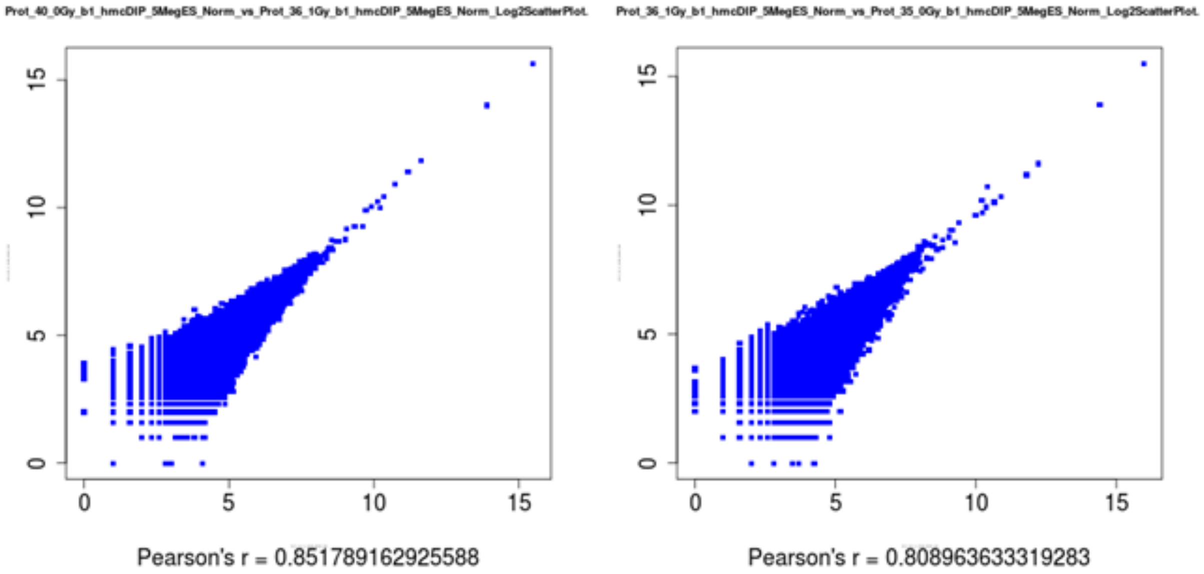

Suppl Fig 3

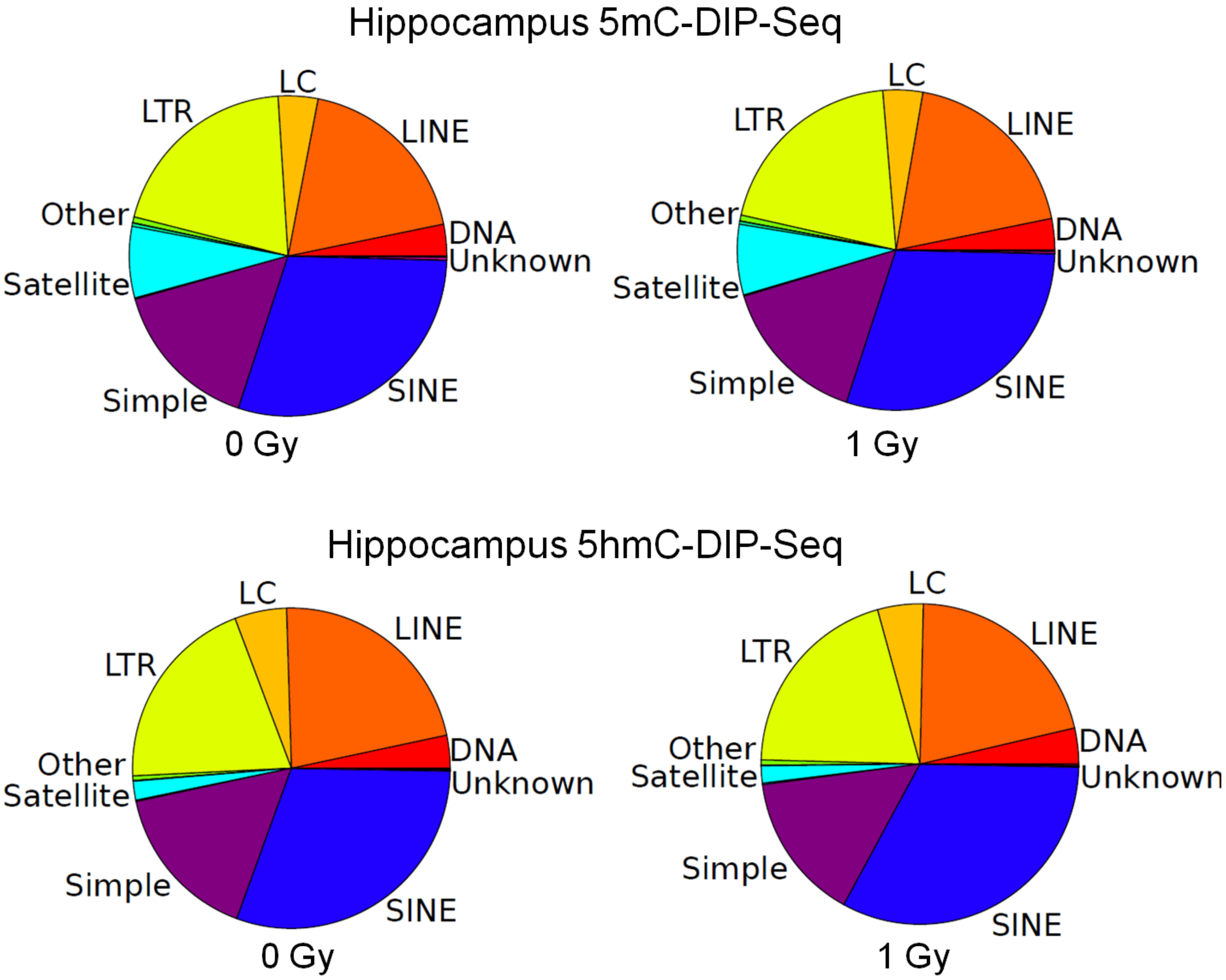
Hippocampus 5mC-DIP-Seq
LC
LTR
LINE
Other
DNA
Unknown
Satellite
Simple
SINE
0 Gy
LC
LTR
LINE
Other
DNA
Unknown
Satellite
Simple
SINE
1 Gy
Hippocampus 5hmC-DIP-Seq
LC
LINE
LTR
DNA
Other
Satellite
Unknown
Simple
SINE
0 Gy
LC
LINE
LTR
DNA
Other
Satellite
Unknown
Simple
SINE
1 Gy

Suppl Fig 4

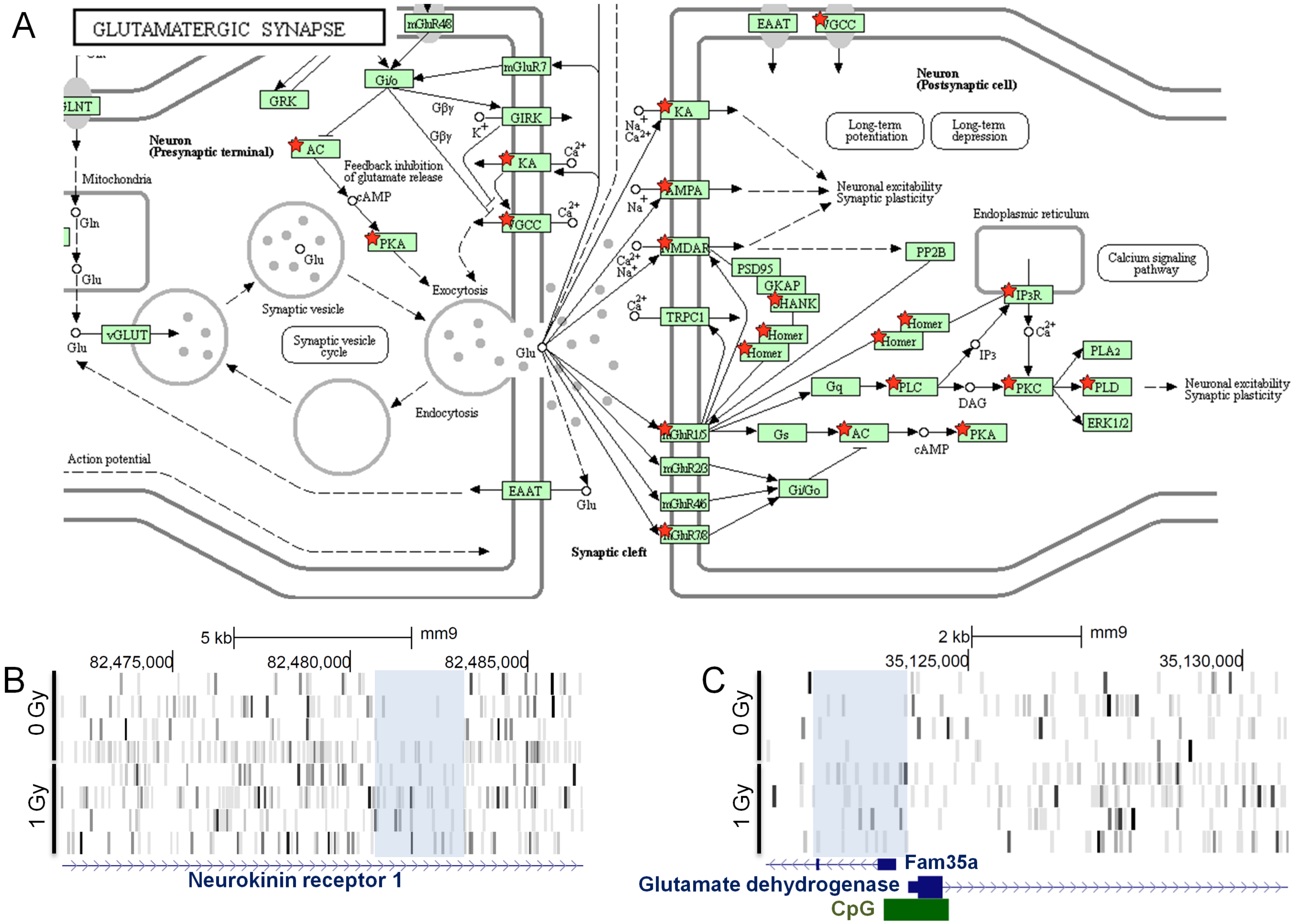
A
GLUTAMATERGIC SYNAPSE
Neuron
(Presynaptic terminal)
Mitochondria
Gln
Glu
vGLUT
GRK
Gi/o
mGluR48
mGluR7
GIRK
KA
VGCC
AC
cAMP
PKA
Feedback inhibition
of glutamate release
Synaptic vesicle
Synaptic vesicle cycle
Exocytosis
Endocytosis
Action potential
EAAT
Synaptic cleft
Neuron
(Postsynaptic cell)
Long-term potentiation
Long-term depression
Neuronal excitability
Synaptic plasticity
AMPA
NMDAR
TRPC1
PSD95
GKAP
SHANK
Homer
PP2B
Endoplasmic reticulum
Calcium signaling pathway
IP3R
IP3
PLC
Gq
DAG
PKC
PLA2
PLD
ERK1/2
mGluR1/5
mGluR2/3
mGluR4/6
mGluR7/8
Gs
Gi/Go
B
0 Gy
1 Gy
5 kb
mm9
82,475,000
82,480,000
82,485,000
Neurokinin receptor 1
C
2 kb
35,125,000
35,130,000
Fam35a
Glutamate dehydrogenase
CpG

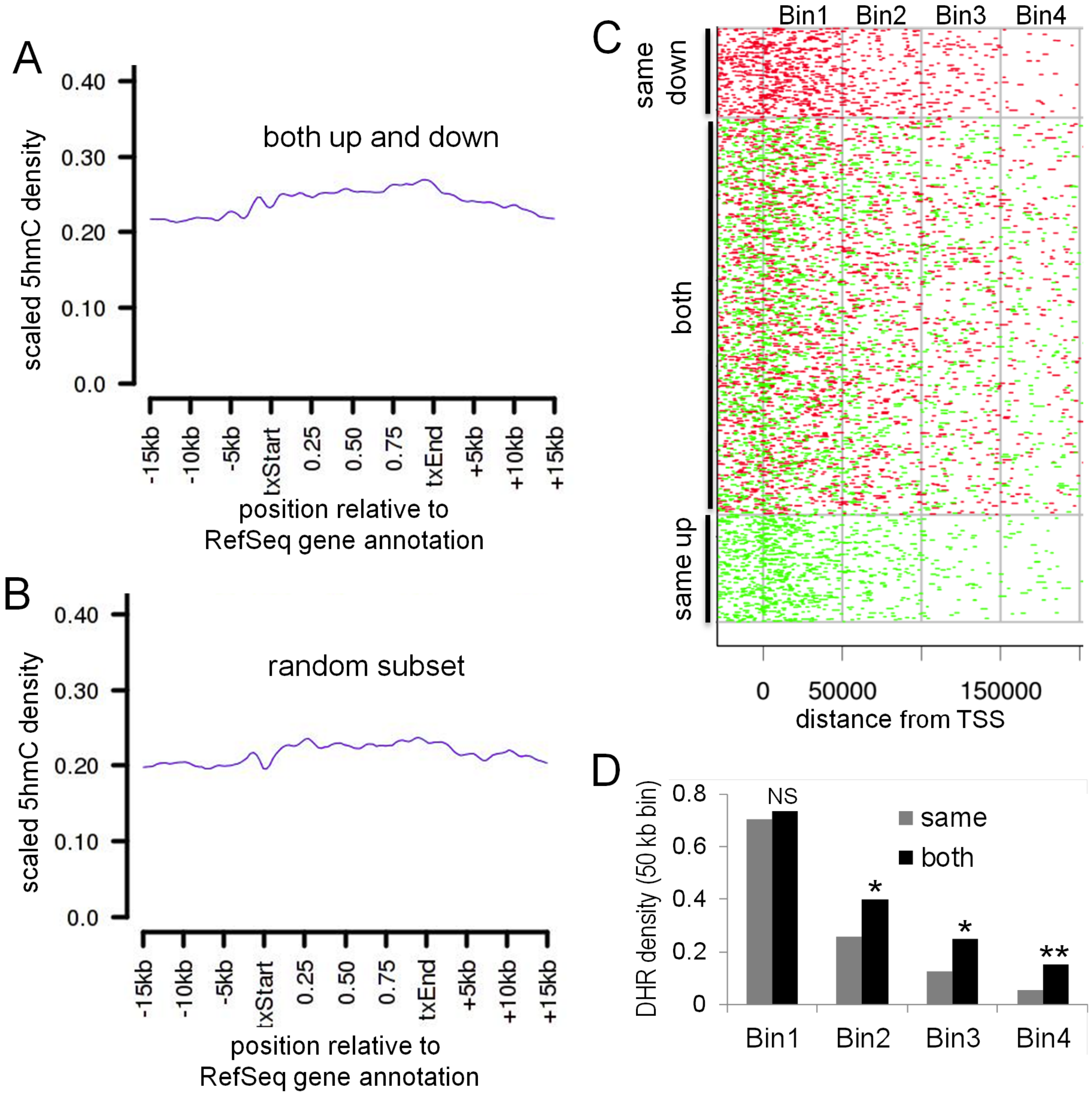
A
0.40
0.30
0.20
0.10
0.0
scaled 5hmC density
both up and down
-15kb
-10kb
-5kb
txStart
0.25
0.50
0.75
txEnd
+5kb
+10kb
+15kb
position relative to
RefSeq gene annotation
B
0.40
0.30
0.20
0.10
0.0
scaled 5hmC density
random subset
-15kb
-10kb
-5kb
txStart
0.25
0.50
0.75
txEnd
+5kb
+10kb
+15kb
position relative to
RefSeq gene annotation
C
Bin1
Bin2
Bin3
Bin4
same down
both
same up
0
50000
150000
distance from TSS
D
DHR density (50 kb bin)
0.8
0.6
0.4
0.2
0
NS
*
*
**
same
both
Bin1
Bin2
Bin3
Bin4

Suppl Fig 6

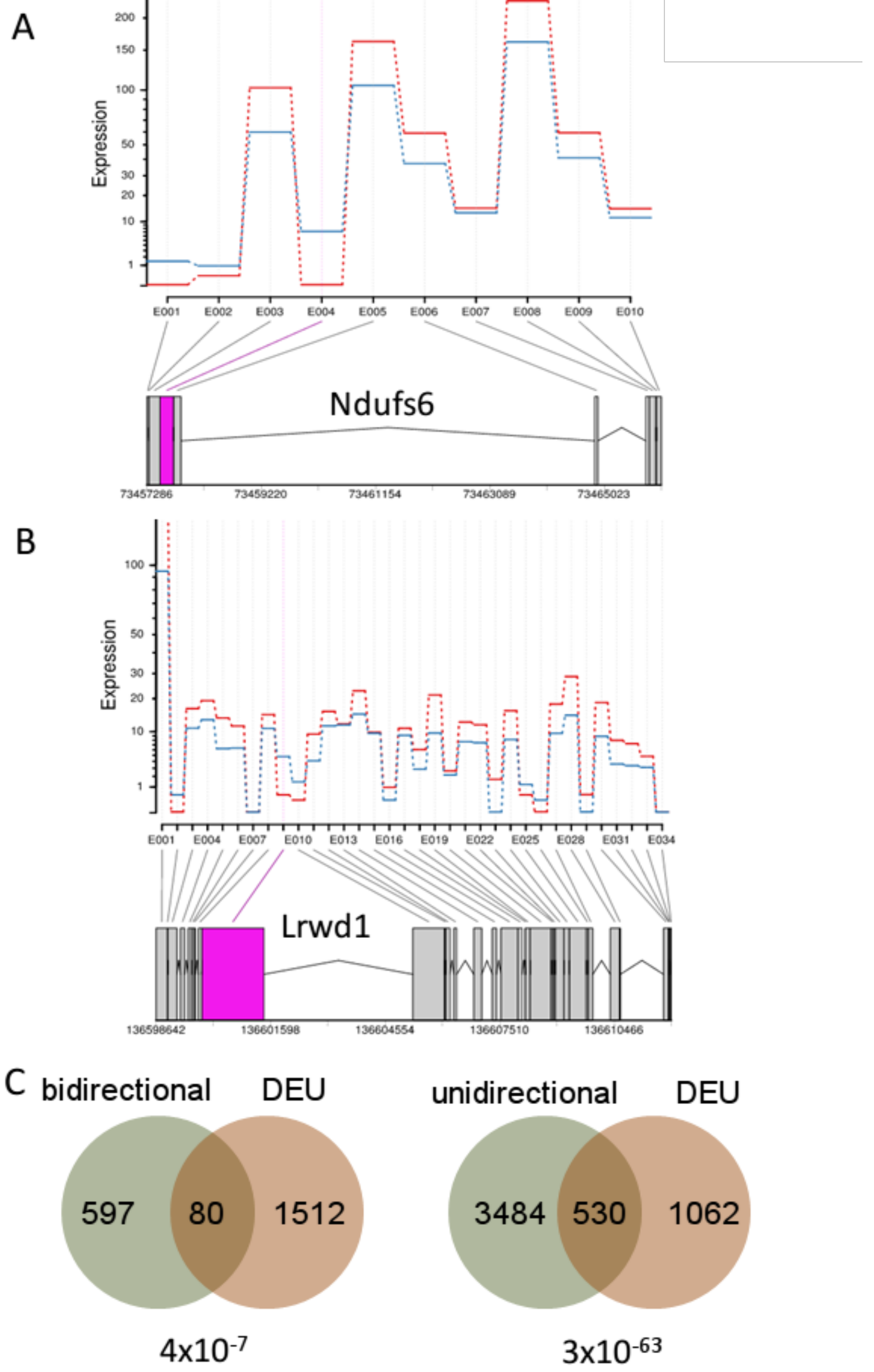
A
Expression
E001
E002
E003
E004
E005
E006
E007
E008
E009
E010
Ndufs6
73457286
73459220
73461154
73463089
73465023
B
Expression
E001
E004
E007
E010
E013
E016
E019
E022
E025
E028
E031
E034
Lrwd1
136598642
136601598
136604554
136607510
136610466
C
bidirectional
DEU
597
80
1512
4x10-7
unidirectional
DEU
3484
530
1062
3x10-63

Suppl Fig 7

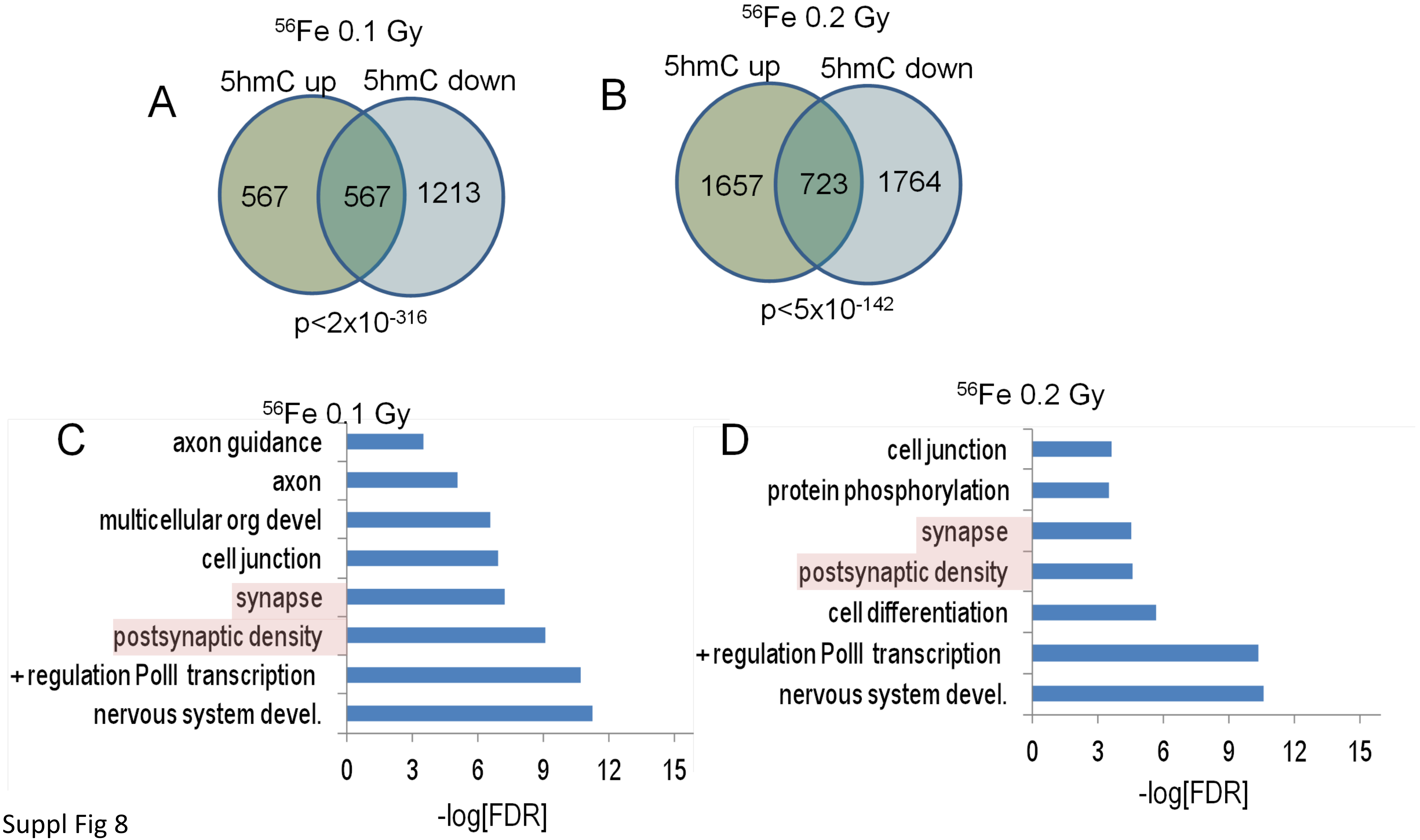
A
56Fe 0.1 Gy
5hmC up
5hmC down
567
567
1213
p<2x10-316
B
56Fe 0.2 Gy
5hmC up
5hmC down
1657
723
1764
p<5x10-142
C
56Fe 0.1 Gy
axon guidance
axon
multicellular org devel
cell junction
synapse
postsynaptic density
+ regulation PolII transcription
nervous system devel.
0
3
6
9
12
15
-log[FDR]
D
56Fe 0.2 Gy
cell junction
protein phosphorylation
synapse
postsynaptic density
cell differentiation
+ regulation PolII transcription
nervous system devel.
0
3
6
9
12
15
-log[FDR]

Suppl Fig 8

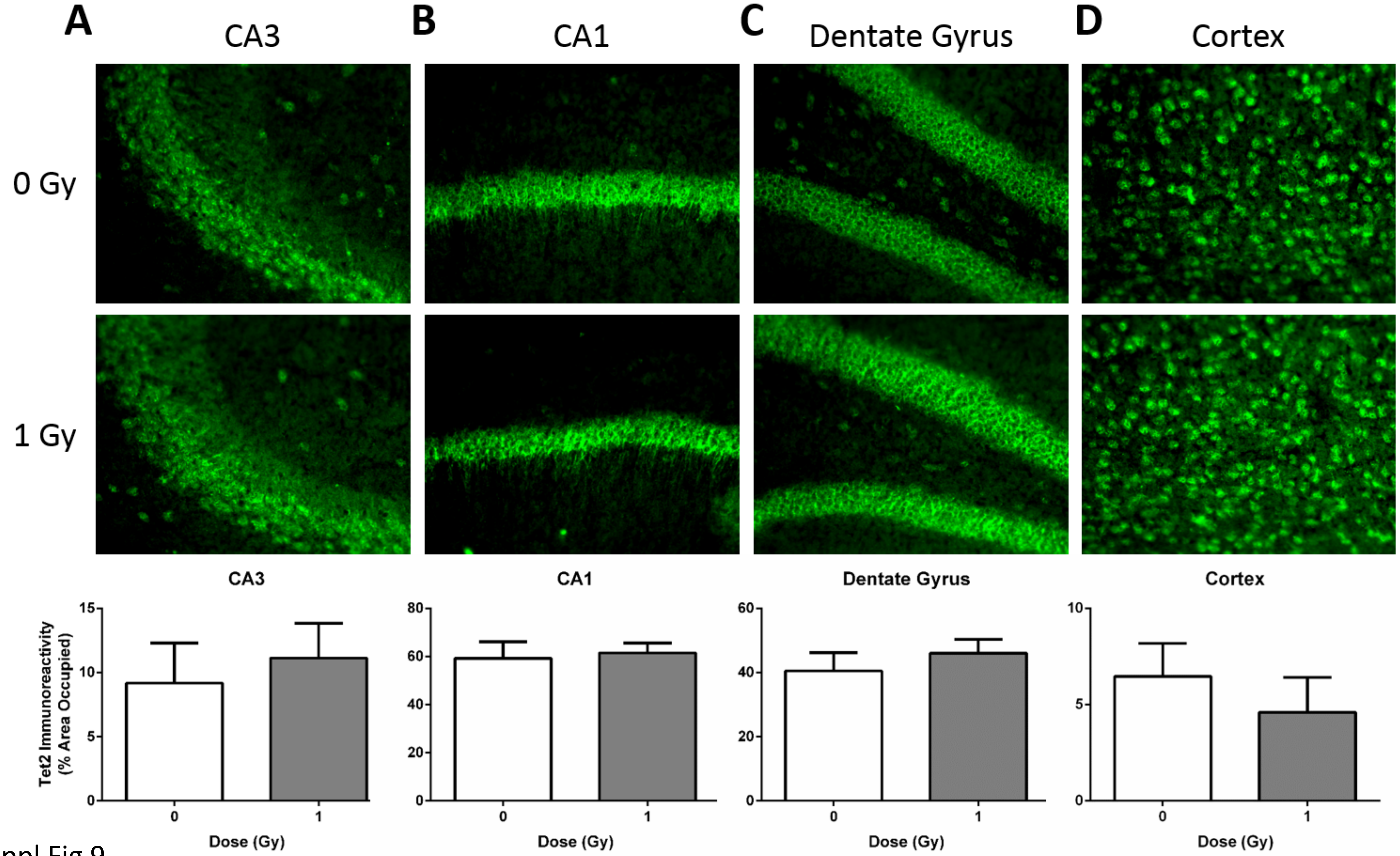
A
CA3
B
CA1
C
Dentate Gyrus
D
Cortex
0 Gy
1 Gy
CA3
CA1
Dentate Gyrus
Cortex
Tet2 Immunoreactivity (% Area Occupied)
15
10
5
0
80
60
40
20
60
10
0
1
Dose (Gy)

Suppl Fig 9

Suppl. Figure Legends

Suppl. Fig. 1. RNA-Seq saturation curves at depth of 5 reads per annotated gene.

Suppl. Fig. 2. (A) Pearson correlation matrix of log2-transformed RNA-Seq exonic reads. The color scale shows Pearson's *r*. (B) Pearson correlation matrix of 5mC- and 5hmC-DIP-Seq reads from indicated samples. Reads were selected from regions of enrichment (FDR-adjusted $p < 0.001$) merged across all samples.

Suppl. Fig. 3. Example correlation scatter plots for some of the comparisons in Suppl. Fig. 2B.

Suppl. Fig. 4. Both 5mc and 5hmC regions are enriched at major parasitic repeats (LINEs, SINEs, LTR-based repeated) with 5mC showing the expected enrichment at Satellite repeats.

Suppl. Fig. 5. KEGG analyses revealed that synapse pathways are significantly associated with increased DHRs. The KEGG database was developed by the Kanehissa Laboratories, as described [72].

Suppl. Fig. 6. A and B. Similar global 5hmC density levels at genes with overlapping up and down DHRs versus a random subset of up alone or down alone. C. Heatmap shows up (green)

and down (red) DHRs from a subset of genes that are >200 kb in genomic length, have two up (same up), two down (same down), or an up and down (both) DHR, and are then randomly sorted by the directionality of intragenic DHRs (horizontal grey line). The indicated bins (grey lines) delineate 50 kb regions distal to the transcriptional start site (TSS). D. A permutation statistic identifies a highly significant increase in DHR density in distal bins for genes with "both" versus genes with "same" DHR distributions (see C). *$p < 1x10^{-4}$ and **$p < 2x10^{-7}$.

Suppl. Fig. 7. Differential exon usage anlaysis. A and B. Gene ideograms depict significantly regulated genes with exon expression levels for 0 Gy and 1 Gy indicated by the red and blue lines respectively. The significantly-regulated exon is highlighted. C. Venn diagram depicts intersection of 677 bidirectional DHRs and 4014 unidirectional DHRs with annotated differentially-expressed exons ($p < 0.01$). The fisher exact test was used to assess significance.

Suppl. Fig. 8. Reanalyzing the ^{56}Fe data using a similar bioinformatics pipeline as used in the current study, we once again observed a strikingly significant overlap between increased and decreased DHRs at the same genes for both the 0.1 Gy and 0.2 Gy doses.

Suppl. Fig. 9. Comparable Tet2 immunoreactivity in sham-irradiated and proton irradiated mice at the 2-week time point. **A.** Tet2 immunoreactivity levels in the CA3 region of the hippocampus. *$p < 0.05$ versus sham-irradiation. **B.** Tet2 immunoreactivity levels in the CA1

region of the hippocampus. **C.** Tet2 immunoreactivity levels in the dentate gyrus. **D.** Tet2 immunoreactivity levels in the cortex. $N = 20$ mice/dose.
